# Lymphocyte crosstalk is required for monocyte-intrinsic trained immunity to *Plasmodium falciparum*

**DOI:** 10.1172/JCI139298

**Published:** 2022-06-01

**Authors:** Juliet N. Crabtree, Daniel R. Caffrey, Leandro de Souza Silva, Evelyn A. Kurt-Jones, Katherine Dobbs, Arlene Dent, Katherine A. Fitzgerald, Douglas T. Golenbock

**Affiliations:** 1 Program in Innate Immunity and; 2 Division of Infectious Diseases and Immunology, Department of Medicine, University of Massachusetts Chan Medical School, Worcester, Massachusetts, USA.; 3 Case Western University, Cleveland, Ohio, USA.; 4 Division of Innate Immunity, Department of Medicine, University of Massachusetts Chan Medical School, Worcester, Massachusetts, USA.

**Keywords:** Immunology, Infectious disease, Innate immunity, Malaria, T cells

## Abstract

*Plasmodium falciparum* (*P. falciparum*) induces trained innate immune responses in vitro, where initial stimulation of adherent PBMCs with *P. falciparum*–infected RBCs (iRBCs) results in hyperresponsiveness to subsequent ligation of TLR2. This response correlates with the presence of T and B lymphocytes in adherent PBMCs, suggesting that innate immune training is partially due to adaptive immunity. We found that T cell–depleted PBMCs and purified monocytes alone did not elicit hyperproduction of IL-6 and TNF-α under training conditions. Analysis of *P. falciparum–*trained PBMCs showed that DCs did not develop under control conditions, and IL-6 and TNF-α were primarily produced by monocytes and DCs. Transwell experiments isolating purified monocytes from either PBMCs or purified CD4^+^ T cells, but allowing diffusion of secreted proteins, enabled monocytes trained with iRBCs to hyperproduce IL-6 and TNF-α after TLR restimulation. Purified monocytes stimulated with IFN-γ hyperproduced IL-6 and TNF-α, whereas blockade of IFN-γ in *P. falciparum*–trained PBMCs inhibited trained responses. Assay for transposase-accessible chromatin with high-throughput sequencing (ATAC-Seq) on monocytes from patients with malaria showed persistently open chromatin at genes that appeared to be trained in vitro. Together, these findings indicate that the trained immune response of monocytes to *P. falciparum* is not completely cell intrinsic but depends on soluble signals from lymphocytes.

## Introduction

*Plasmodium falciparum* (*P. falciparum*) remains one of the deadliest diseases in the world today. In 2020, WHO reported that there were an estimated 241 million cases of malaria that took the lives of 627,000 individuals — most of these were children under the age of 5 (1). The features of the acute uncomplicated disease include high-spiking fevers, profound malaise, headache, and other systemic signs of inflammation (2–4). These symptoms suggest that parasite products (pathogen-associated molecular patterns) engage the myeloid compartment, activating a panel of innate immune sensors that drive cytokine production and inflammation (5).

Patients with malaria have an increased risk of developing invasive bacterial infection, and these bacterial coinfections substantially increase the risk of mortality (6, 7). A longitudinal study conducted in Kenya found that greater than 50% of all the bacteremia cases in the region were attributable to malaria infection (8). The mechanisms underlying this increased susceptibility to bacteremia are not fully understood. Researchers have hypothesized that acute malaria infection primes innate inflammatory pathways, including the TLR and NLR pathways (3, 9), such that upon sensing bacterial pathogens, they produce a hyperinflammatory response that is detrimental to the host. Indeed, in a murine model of malaria and bacterial coinfection, it was demonstrated that acute *Plasmodium* infection induces high levels of pro–caspase-1 in monocytes, which resulted in elevated levels of activated caspase-1 and extraordinarily high levels of the proinflammatory cytokine IL-1β after bacterial infection, culminating in a dramatic increase in mortality (9). These data suggest that during acute malaria infection, the innate immune system is poised for a hyperinflammatory response to subsequent bacterial insults.

Numerous studies have demonstrated a link between bacterial infection occurring shortly after febrile malaria and a fatal outcome (7). This observation suggests that epigenetic changes in the response of phagocytic leukocytes to bacteria resulting in hyperinflammation lead to complications of malaria infection, including cerebral malaria, and hence are an important cause of death. Indeed, there are reports of enhanced priming and inflammatory responses in monocytes of patients with acute malaria, prompting the serious consideration that immune training could be occurring (3, 10). Certainly, an understanding of the basis of hyperinflammation during malaria due to innate immune training is worthwhile (7, 11) given that strategies to recognize and control for this event do not yet exist.

To study this hyperinflammatory response, we and others have focused on experimental designs that explore the mechanisms of innate immune cell activation and how they are poised to hyperrespond to subsequent inflammatory ligands (12–15). Here, we describe a monocyte-centric view of this process, and we showed that monocyte-extrinsic signals were essential to induce a monocyte-intrinsic amplification of TLR-mediated inflammation. These findings were surprising because the literature suggests that “purified” monocytes can mount an amplified inflammatory response (12–14). Technical differences might account for some of the confusion as we found that simple monocyte purification techniques that are widely used in the literature, especially the adherence to tissue culture plastic, were poorly efficacious. Indeed, monocytes purified to near homogeneity using more modern techniques lost their ability to hyperrespond to TLR ligation. This led us to hypothesize that lymphocyte crosstalk with monocytes is involved in innate immune training in response to *P*. *falciparum* stimulation. Furthermore, exposure to *P*. *falciparum* induced a marked change in the populations of immune cells in PBMCs, with a robust representation of cytokine-producing DCs and monocyte populations.

## Results

### P. falciparum exposure amplifies the inflammatory response to a TLR stimulus in adherent PBMCs but not in highly purified monocytes.

**Figure 1:**
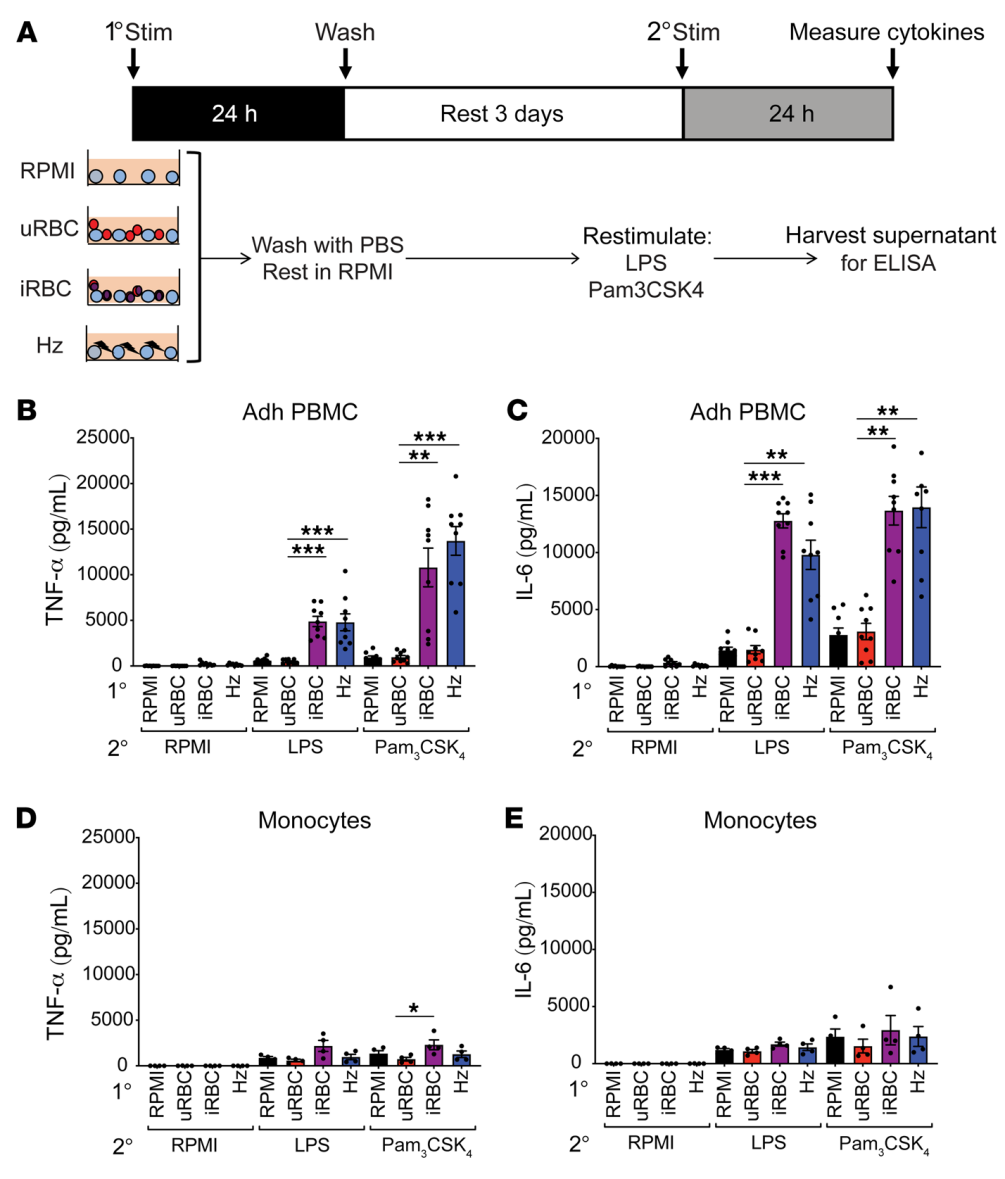
*P*. *falciparum* induces hyperinflammation in adherent PBMCs but not purified monocytes. (**A**) Schematic of the in vitro experimental design. TNF-α (**B**) and IL-6 (**C**) ELISAs of adherent (Adh) PBMCs after primary stimulation with RPMI medium, 1 × 10^6^ uninfected erythrocytes (uRBCs), 1 × 10^6^ *P*. *falciparum*–infected erythrocytes (iRBCs), or 100 μM hemozoin (Hz); rested 3 days in medium; and restimulated with RPMI medium, 10 ng/mL LPS, or 10 μg/mL Pam_3_CSK_4_ for 24 hours. *n =* 9; data shown as t+he mean ± SEM. TNF-α (**D**) and IL-6 (**E**) ELISAs of supernatants from purified monocytes after primary stimulation with RPMI, 1 × 10^6^ uRBCs, 1 × 10^6^ iRBCs, or 50 μM Hz; rested 3 days in medium; and restimulated with RPMI medium, 10 ng/mL LPS, or 10 μg/mL Pam_3_CSK_4_ for 24 hours. *n =* 4; data shown as mean ± SEM. *P ≤ 0.05, **P ≤ 0.01, ***P ≤ 0.001, by Kruskal-Wallis nonparametric ANOVA with Dunn’s multiple-comparison test.

Previous work from our lab revealed that an initial exposure to *P*. *falciparum–*infected RBCs (iRBCs) or the natural malaria crystal hemozoin (Hz) induced human adherent PBMCs to hyperrespond to subsequent ligation of TLR2, even if the second exposure was several days after the first (15). Consistent with these findings, adherent PBMCs given a primary exposure to *P*. *falciparum* iRBCs or Hz hyperproduced TNF-α and IL-6 upon restimulation with the TLR agonists LPS or Pam_3_CSK_4_ — unlike adherent PBMCs first treated with RPMI medium or uninfected erythrocytes (uRBCs) (Figure 1, B and C). To further study this process, we employed magnetic-assisted cell sorting (MACS) to generate highly purified monocytes, which were then incubated with RPMI medium, uRBCs, iRBCs, or Hz and restimulated with RPMI medium, LPS, or Pam_3_CSK_4_. To our surprise, the purified monocytes failed to hyperproduce TNF-α and IL-6 when subjected to this treatment (Figure 1, D and E). This was surprising because adherence purification of PBMCs has been widely used to enrich for monocytes in trained immunity models (12, 13, 16), and these studies attribute the hyperproduction of proinflammatory cytokines as a monocyte-intrinsic mechanism. This observation indicated that purified monocytes alone were incapable of undergoing innate immune training and that other cell types were required for this phenomenon.

### Recent exposure of PBMCs to P. falciparum leads to a sustained immune response that is further amplified by PAM_3_CSK_4_.

Next, we performed RNA-Seq in PBMCs to investigate whether recent exposure to *P*. *falciparum* also led to an enhanced global transcriptional response after PAM_3_CSK_4_ stimulation. Similar to the experiments described above (Figure 1A), we incubated PBMCs with either RPMI medium, uRBCs, or iRBCs for 1 day and then rested them for 3 days prior to PAM_3_CSK_4_ stimulation. We stimulated cells with PAM_3_CSK_4_ for 0, 4, and 12 hours before quantifying global RNA expression. We focused on comparing PBMCs recently incubated with iRBCs to those incubated with uRBCs (control) because the samples with RPMI medium and uRBCs had highly correlated expression levels (Supplemental Figure 1A; supplemental material available online with this article; https://doi.org/10.1172/JCI139298DS1).

**Figure 2:**
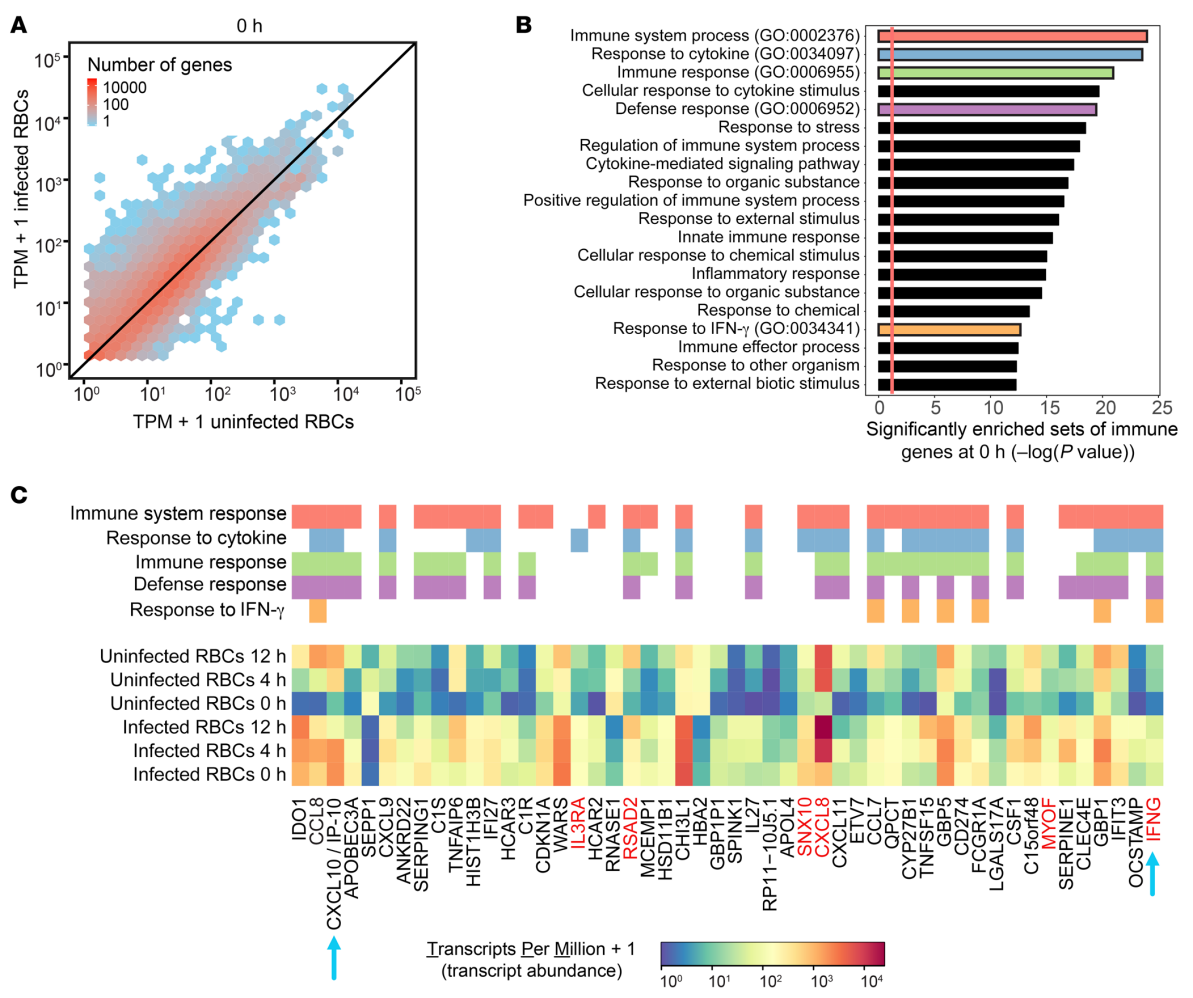
Recent exposure of PBMCs to *P*. *falciparum* leads to a sustained immune response that is further amplified by PAM_3_CSK_4_. (**A**) RNA expression of PBMCs recently exposed to infected and uninfected RBCs and subsequently stimulated with PAM_3_CSK_4_ (after 0 hours). RNA expression units correspond to transcripts per million (TPM) mapped reads. Each hexagonal bin shows the number of genes with the corresponding expression level. *n =* 3. (**B**) Significantly enriched GO terms (biological processes) among the 100 genes with the greatest fold change between PBMCs recently exposed to infected RBCs and PBMCs exposed to uninfected RBCs at 0 hours prior to PAM_3_CSK_4_ stimulation. Five GO terms of interest (red, blue, green, purple, and orange) were further examined in **C**. (**C**) Expression levels (TPM) among the 50 genes with the greatest fold change between PBMCs recently exposed to infected RBCs and PBMCs exposed to uninfected RBCs at 0 hours prior to PAM_3_CSK_4_ stimulation. Expression levels at 4 and 12 hours after PAM_3_CSK_4_ stimulation are also shown. Genes belonging to the 5 GO terms of interest (red, blue, green, purple, and orange) are indicated above the heatmap. The blue arrows indicate genes that are described in the main text. The red gene labels indicate genes that are shown in a subsequent ATAC-Seq experiment.

Prior to PAM_3_CSK_4_ stimulation (0 hours), global mRNA levels were already higher in PBMCs incubated with iRBCs than uRBCs (Figure 2A). Among the genes that were significantly altered over the time course for PBMCs incubated with iRBCs and/or uRBCs, there were 1068 genes with a 3-fold or greater difference in expression at 0 hours (Supplemental Figure 1B). We rank-ordered these 1068 genes by absolute fold change and performed a gene ontology (GO) enrichment analysis on the top 100 genes, which revealed significantly enriched sets of immune response genes (Figure 2B). The top 50 genes included *CXCL10/IP-10* and *IFNG*, as well as other genes that respond to IFN-γ (Figure 2C). Among these 50 genes, expression levels tended to be higher in PBMCs exposed to iRBCs than to uRBCs at all time points. Although the temporal expression patterns varied according to the gene, PAM_3_CSK_4_ tended to amplify gene expression in PBMCs that were exposed to iRBCs. Taken together, these results indicated that recent exposure of PBMCs to *P*. *falciparum* led to a sustained immune response that was further amplified by PAM_3_CSK_4_.

### Adherent PBMCs contain lymphocyte contaminants, but myeloid cells are the main producers of TNF-α and IL-6.

**Figure 3:**
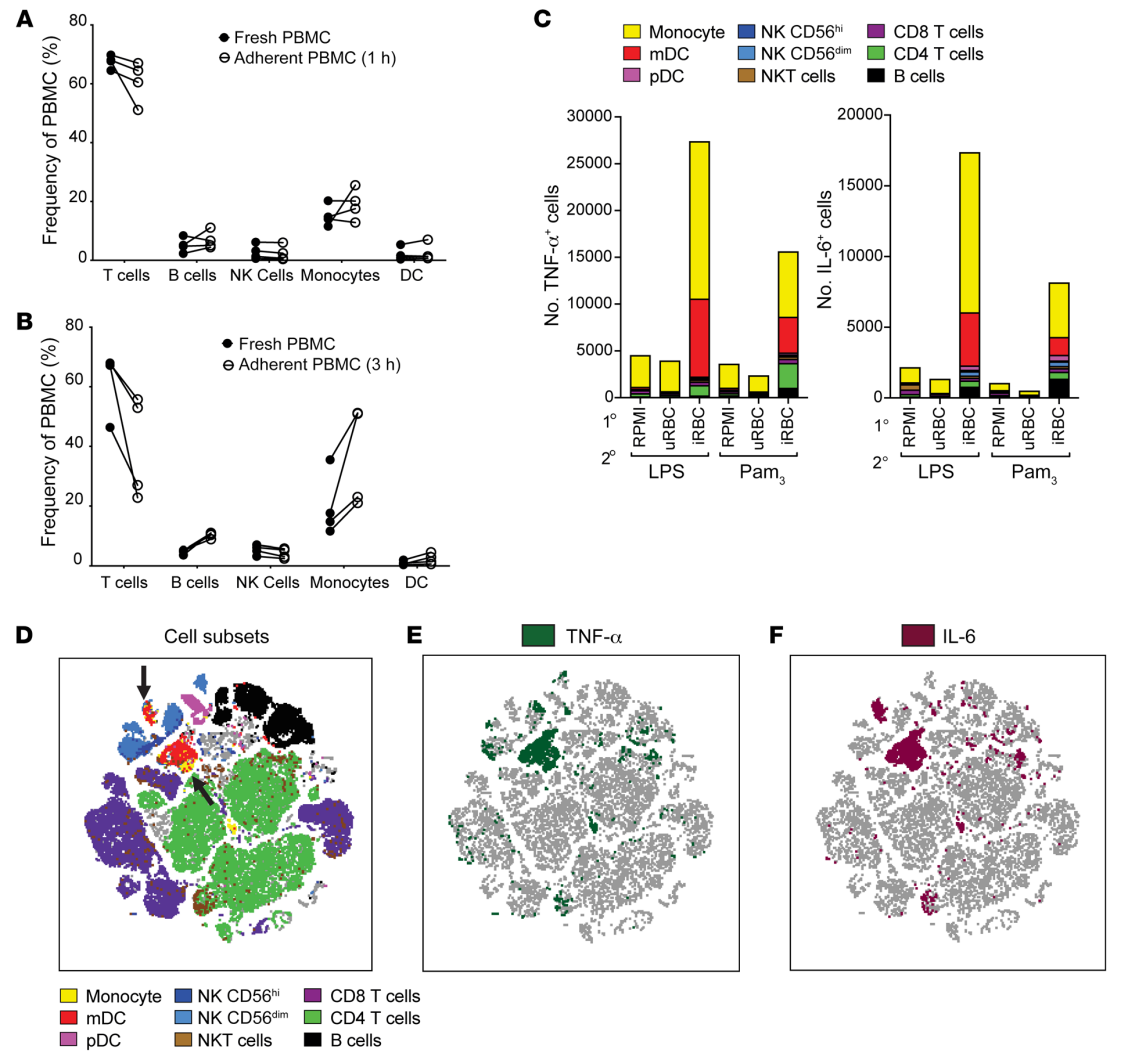
Myeloid cells are the main proinflammatory cytokine producers of *P.* *falciparum*–trained PBMCs. (**A** and **B**) Flow cytometry analysis indicating cell subset frequency of freshly isolated PBMCs and PBMCs that had adhered to tissue culture plates for 1 hour (**A**) or 3 hours (**B**) before 3 successive washes with PBS (*n =* 4). (**C**) Intracellular cytokine staining of TNF-α and IL-6 for PBMCs trained with the indicated primary stimulus, rested 3 days in medium, and restimulated with 10 ng/mL LPS or 10 μg/mL Pam_3_CSK_4_ for 5 hours (*n =* 4). (**D**–**F**) tSNE plot of all combined samples in **C**. (**D**) Colored overlays indicate individual cell types depicted in the legend. Black arrows indicate populations of myeloid cells (monocytes and DCs). (**E**) Overlay of all TNF-α^+^ cells (dark green). (**F**) Overlay of all IL-6^+^ cells (crimson). mDC, myeloid DC; pDC, plasmacytoid DC.

Having defined the impact of *P*. *falciparum* training on the transcriptome of PBMCs, we next wanted to define the cells in the PBMC population that were being trained. Therefore, we analyzed the cellular components of fresh PBMCs and adherent PBMCs that had adhered for 1 hour or 3 hours before 3 subsequent PBS washes and found that adherence purification left ample lymphocyte contaminants. After 1 hour of adherence, the adherent PBMC cellular components looked almost identical to fresh PBMCs (Figure 3A), and for the 3-hour adherence, the enrichment for monocytes ranged from 20% to 50% (Figure 3B). This finding raised concerns about whether monocytes were in fact the cells being trained and responsible for producing TNF-α and IL-6, or whether other lymphocyte subsets could be producing large amounts of these cytokines. To address this, we performed intracellular cytokine staining for TNF-α and IL-6 using a panel of antibodies against cellular markers that identified 9 different cell types (Supplemental Figure 2) on PBMCs that were trained with either RPMI medium, uRBCs, or iRBCs and then restimulated with LPS or Pam_3_CSK_4_ for 5 hours. We found that there were minimal TNF-α^+^ or IL-6^+^ cells in the RPMI and uRBC training conditions, and that there was a large increase in the number of TNF-α^+^ and IL-6^+^ cells for the iRBC training condition (Figure 3C). We also discovered that, of the total TNF-α^+^ and IL-6^+^ cells, monocytes (CD14^+^HLA-DR^hi^CD68^+^) and myeloid DCs (mDCs; CD14^–^HLA-DR^hi^CD11c^+^) were the most abundant producers (Figure 3, C–F), with smaller contributions from CD4^+^ T cells and B cells for TNF-α and IL-6, respectively (Figure 3C). When analyzing the frequency of TNF-α– or IL-6–producing cells relative to total viable cells, both monocytes and mDCs had significantly higher frequencies of TNF-α^+^ or IL-6^+^ cells in iRBC-treated PBMCs than in the RPMI medium or uRBC controls (Supplemental Figure 3, A–D). Together, these findings showed that, although the adherence purification method failed to eliminate lymphocytes, the main cells responsible for hyperproduction of proinflammatory cytokines TNF-α and IL-6 were myeloid cells, specifically monocytes and mDCs.

### Loss of T lymphocytes ablates hyperinflammatory responses in monocytes.

**Figure 4:**
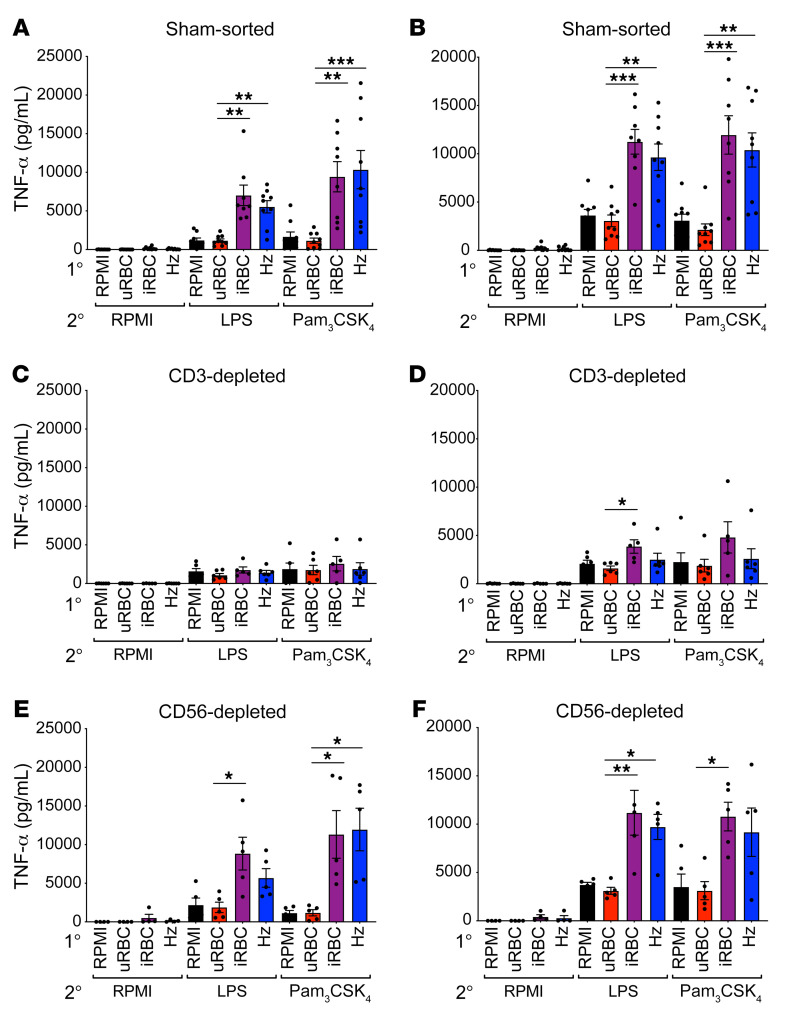
Loss of T lymphocytes ablates the hyperinflammatory response in monocytes. TNF-α and IL-6 ELISAs of cells trained for 24 hours with the indicated primary stimulus; rested 3 days in medium; and then restimulated with RPMI medium, 10 ng/mL LPS, or 10 μg/mL Pam_3_CSK_4_ for 24 hours. (**A** and **B**) Sham-sorted PBMCs; *n =* 8. (**C** and **D**) PBMCs depleted of CD3^+^ cells; *n =* 5. (**E** and **F**) PBMCs depleted of CD56^+^ cells; *n =* 5. Data shown as the mean ± SEM. *P ≤ 0.05, **P ≤ 0.01, ***P ≤ 0.001, by Kruskal-Wallis nonparametric ANOVA with Dunn’s multiple-comparison test.

Highly purified monocytes failed to hyperproduce proinflammatory cytokines when trained with *P*. *falciparum* iRBCs or Hz (Figure 1); however, in trained PBMCs, the major cytokine-producing cells are monocytes and DCs (Figure 3). Therefore, we hypothesized that another cellular component of PBMCs was critical to support monocyte training. T lymphocytes and NK cells are important mediators of immunity during malaria infection by producing cytokines, such as IFN-γ, and also through direct lysis of iRBCs (17–19). Additionally, the RNA-Seq data highlighted the response to IFN-γ in the GO analysis as a pathway that was induced during training (Figure 2). To that end, we tested whether depleting T cells or NK cells from PBMCs affected the ability of monocytes to hyperrespond to TLR stimuli after iRBC training. PBMCs that underwent a sham-sort procedure maintained strong hyperproduction of TNF-α and IL-6 in response to TLR restimulation (Figure 4, A and B), indicating that the MAC-sorting procedure did not inhibit the trained immune response. However, depletion of CD3^+^ T lymphocytes from PBMCs ablated the hyperproduction of TNF-α and IL-6 in iRBC- and Hz-training conditions (Figure 4, C and D). Notably, depletion of CD56^+^ cells from PBMCs, which eliminates all types of NK cells, had no effect on trained immune responses (Figure 4, E and F). Thus, T lymphocytes were required for PBMCs to mount trained immune responses to malaria, whereas the presence of NK cells was dispensable.

### Monocytes require T lymphocytes to help induce a hyperinflammatory response.

**Figure 5:**
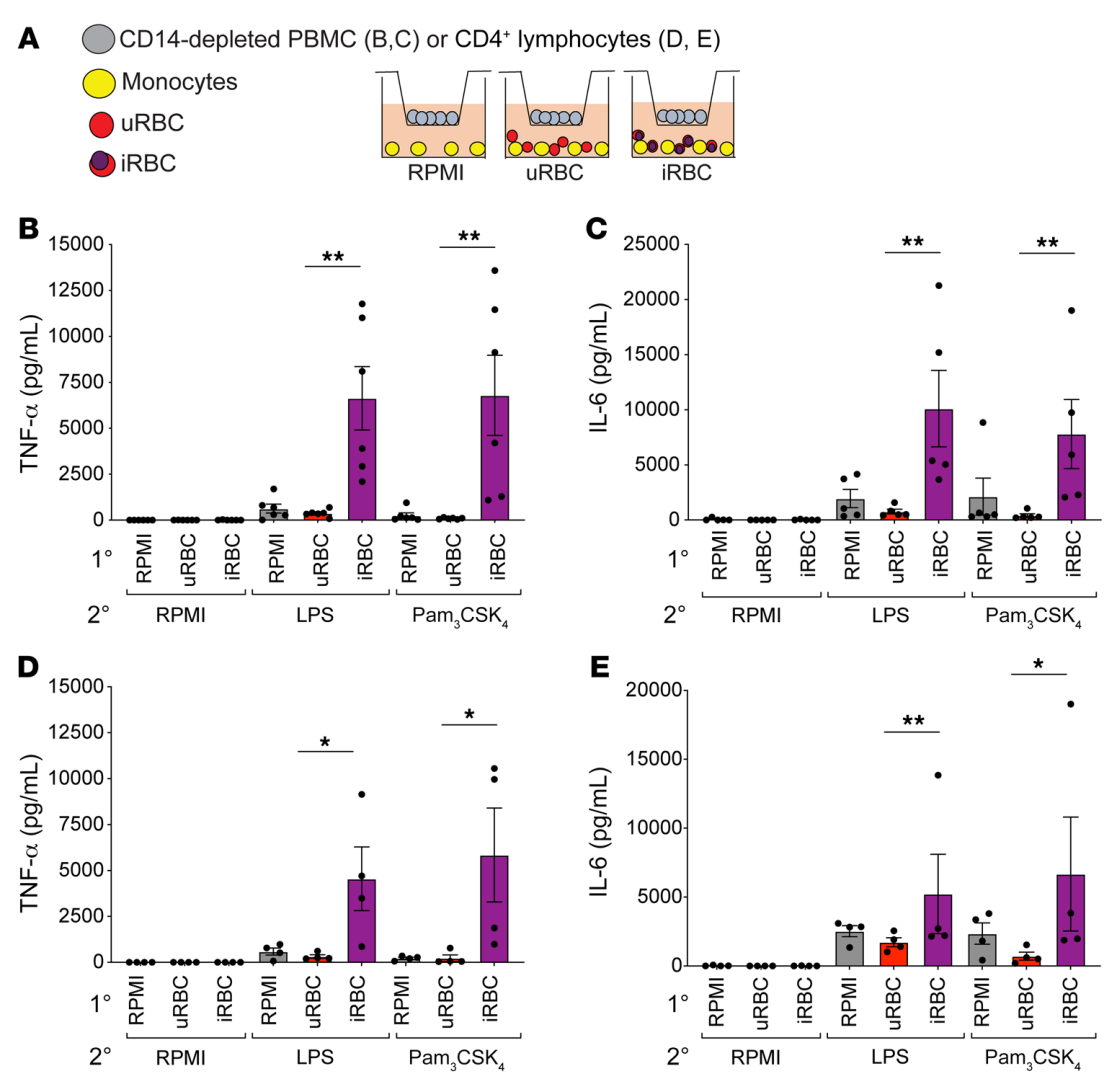
Monocytes require T lymphocytes to induce hyperinflammatory responses. (**A**) Schematic of the Transwell experiment. Purified monocytes (yellow) were plated in a well with the indicated primary stimulus. Lymphocytes were plated in a 0.4 μm pore Transwell above. TNF-α (**B**) and IL-6 (**C**) ELISAs of monocytes stimulated with indicated primary stimuli, with PBMCs depleted of CD14^+^ cells in a Transwell; rested 3 days in medium; and restimulated with RPMI medium, 10 ng/mL LPS, or 10 μg/mL Pam_3_CSK_4_. TNF-α (**D**) and IL-6 (**E**) ELISAs of monocytes stimulated with indicated primary stimuli, with purified CD4^+^ T cells in a Transwell; rested 3 days in medium; and restimulated with RPMI medium, 10 ng/mL LPS, or 10 μg/mL Pam_3_CSK_4_. (**B** and **C**), *n =* 6; (**D** and **E**), *n =* 4. Data shown as mean ± SEM. *P ≤ 0.05, **P ≤ 0.01, by Kruskal-Wallis nonparametric ANOVA with Dunn’s multiple-comparison test.

The inability of highly purified monocytes to mount a trained immune response, and the necessity of CD3^+^ T lymphocytes for PBMCs to mount a trained immune response, suggested that some T cell–derived factor(s) are required to support monocyte training. To test this hypothesis, we employed a Transwell system, where purified monocytes were plated in the bottom well along with the training stimulus (either RPMI medium, uRBCs, or iRBCs), and PBMCs that had been depleted of CD14^+^ monocytes were plated in a Transwell for the duration of the training period and then were removed for the restimulation (Figure 5A). This system allowed for secreted proteins to pass through the Transwell membrane but prevented the cells in the Transwell from directly interacting with the monocytes via cell-cell contact. When monocytes were trained in the presence of CD14-depleted PBMCs and then restimulated with LPS or Pam_3_CSK_4_, we found those that had been trained with iRBCs hyperproduced TNF-α and IL-6 (Figure 5, B and C). Similarly, if we provided purified CD4^+^ T cells in the Transwell during the training period, the monocytes trained with iRBCs were able to hyperproduce TNF-α and IL-6 upon restimulation with LPS or Pam_3_CSK_4_ (Figure 5, D and E). This indicated that soluble factors from T lymphocytes in PBMCs supported monocyte-intrinsic hyperinflammatory responses.

### P. falciparum–trained PBMCs have increased levels of monocytes and DCs.

**Figure 6:**
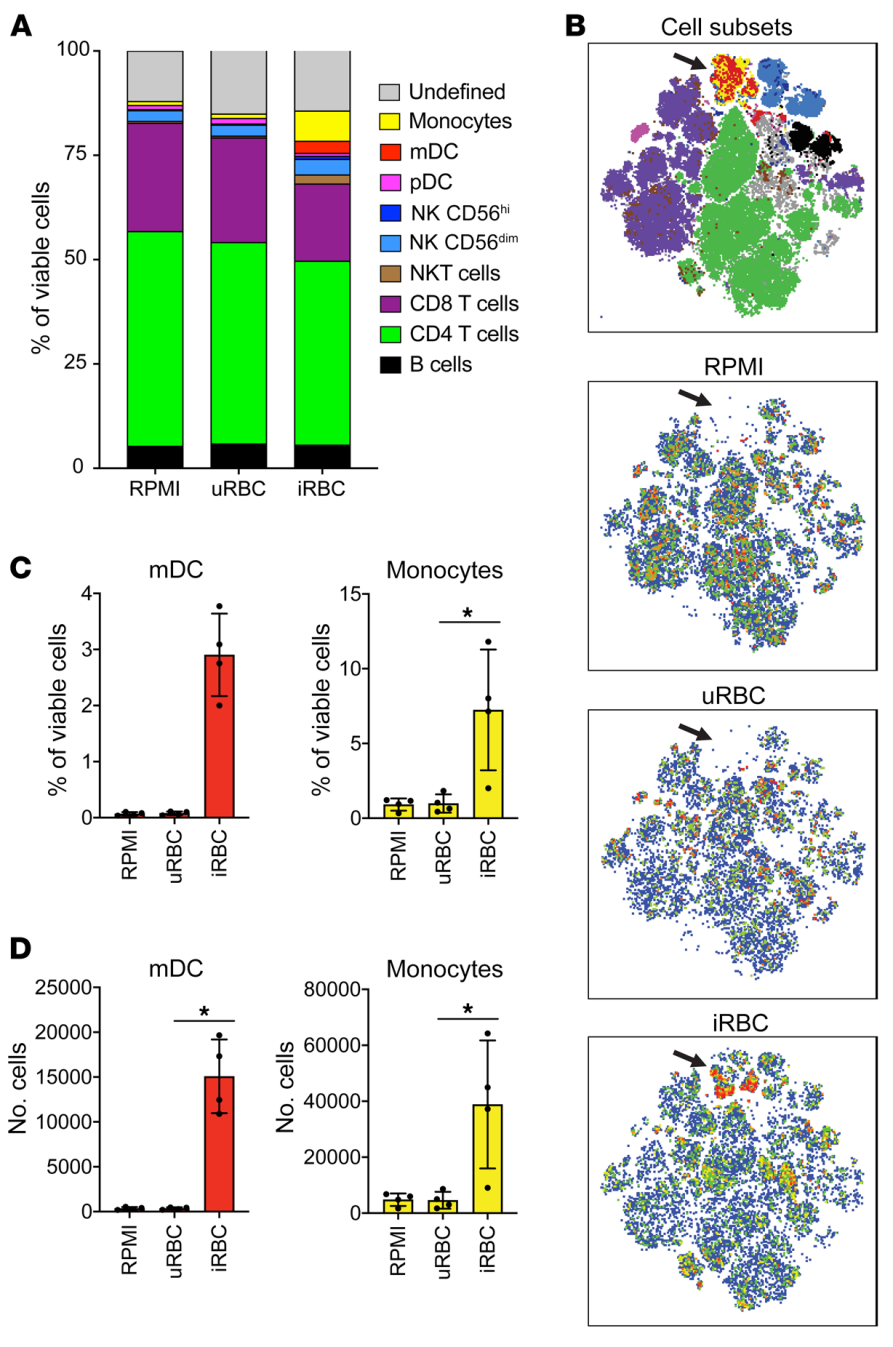
*P*. *falciparum*–trained PBMCs have increased levels of monocytes and DCs. Flow cytometric enumeration of cell subsets in PBMCs stimulated for 24 hours with indicated primary stimuli and rested 3 days in medium. (**A**) Cell type frequency of viable cells. (**B**) tSNE plots showing cell subsets overlaid in color according to the legend in **A** and individual samples trained with RPMI, uRBCs, or iRBCs. Black arrows indicate populations of myeloid cells (monocytes and DCs). (**C**) Frequency of viable cells for mDCs and monocytes for indicated training stimuli. (**D**) Cell number of mDCs and monocytes for indicated training stimuli. *n =* 4; data shown as the mean ± SD. *P ≤ 0.05, by Kruskal-Wallis nonparametric ANOVA with Dunn’s multiple-comparison test.

T lymphocytes produce secreted proteins that modulate monocyte survival, activation, and maturation (20–22). We analyzed the frequency of each cell type present in the trained PBMCs and found that in the RPMI medium and the uRBC training conditions, both monocytes and mDCs made up a very small portion of the total cells (Figure 6, A and B). However, in the iRBC-trained PBMCs, we found a much higher frequency of monocytes and mDCs (Figure 6, A–C) and a higher overall cell number of monocytes and mDCs relative to the RPMI medium and uRBC conditions (Figure 6D). The overrepresentation of monocytes and mDCs in the iRBC-treated PBMCs suggests that these cell types may be receiving prosurvival or antiapoptotic signals, which are absent in the unstimulated conditions. Consistent with this, we saw a marked upregulation of surface programmed death-ligand 1 (PDL1) expression on monocytes and mDCs in the first 12 hours of iRBC stimulation (Supplemental Figure 4). It is known that PDL1 signaling protects cells from apoptosis (23) and that PDL1 expression is positively regulated by IFN-γ (24, 25), a cytokine that is largely produced by T lymphocytes and that we observed was highly expressed in our transcriptome data (Figure 2).

### IFN-γ enhances monocyte training.

**Figure 7:**
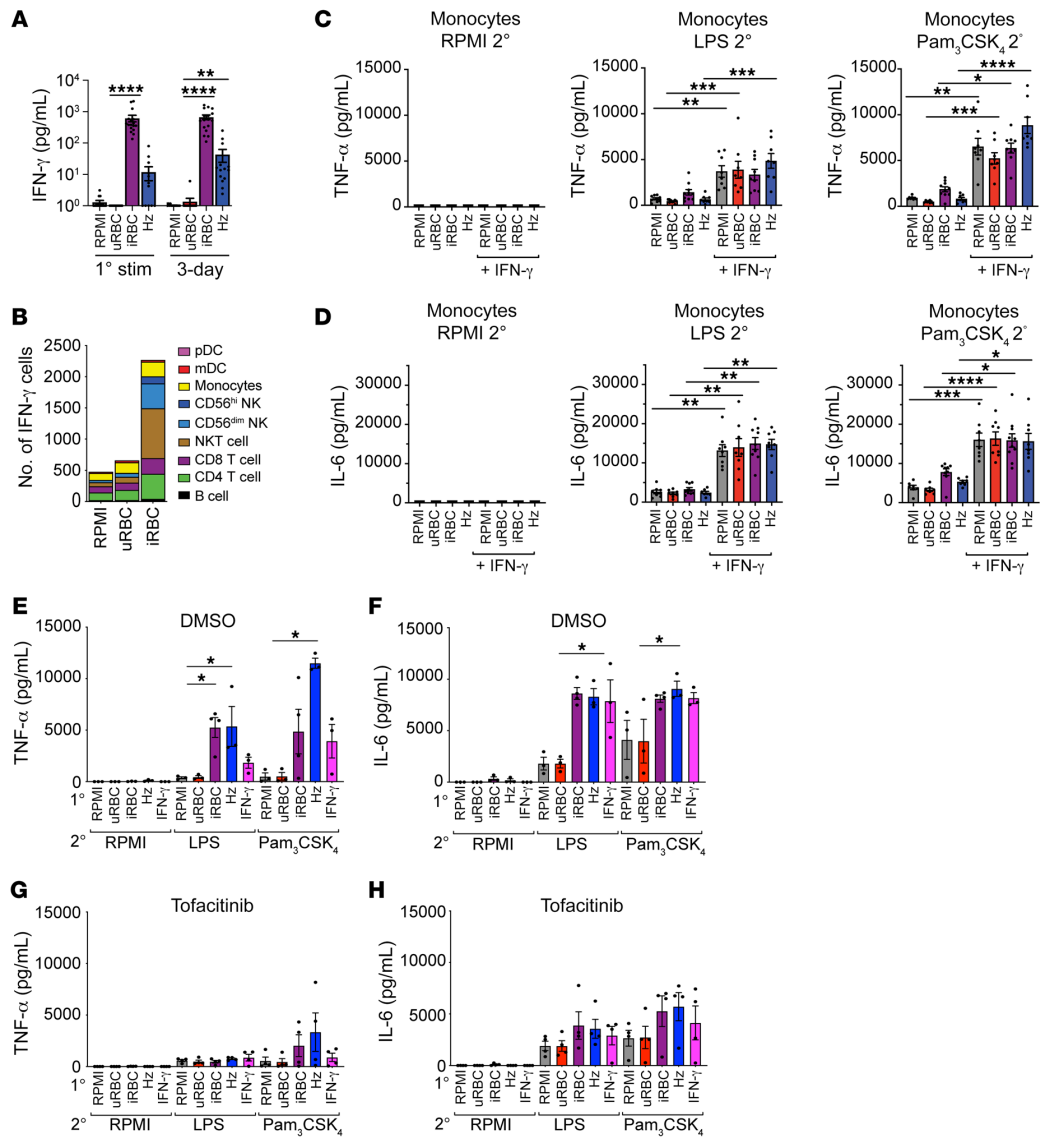
IFN-γ enhances monocyte training. (**A**) ELISA of IFN-γ from supernatants of trained PBMCs during primary stimulus or after a 3-day rest (*n =* 17). (**B**) IFN-γ–producing cells in PBMCs stimulated for 12 hours with the indicated primary stimulus (*n =* 5). TNF-α (**C**) and IL-6 (**D**) ELISAs performed on the supernatant of purified monocytes with or without 20 ng/mL IFN-γ during the training period and with the indicated secondary stimulus. *n =* 8. TNF-α (**E** and **G**) and IL-6 (**F** and **H**) ELISAs from supernatants of trained PBMCs treated with DMSO (**E** and **F**, *n =* 4) or 2 μM of tofacitinib (**G** and **H**, *n =* 4) during 24-hour training and 3-day rest period followed by indicated secondary stimulus. Data shown as mean ± SEM. *P ≤ 0.05, **P ≤ 0.01, ***P ≤ 0.001, ****P ≤ 0.0001, by Kruskal-Wallis nonparametric ANOVA with Dunn’s multiple-comparison test.

Monocytes were unable to mount hyperinflammatory responses after iRBC training without the presence of lymphocytes (Figure 1 and Figure 4), yet the presence of soluble factors from T lymphocytes was sufficient to support monocyte training (Figure 5). Both cytotoxic CD8^+^ T cells and Th1 CD4^+^ T cells are major producers of IFN-γ (26). There were high expression of IFN-γ and an enrichment for IFN-γ signaling in the transcriptome of iRBC-trained PBMCs (Figure 2). IFN-γ is a key factor for the development of trained immunity in response to *Candida albicans* infection (27) and viral lung infection (28). IFN-γ is required for priming the innate immune response in a murine model of malaria (3). We therefore hypothesized that IFN-γ may be sufficient to induce hyperinflammatory responses in monocytes. We found that IFN-γ was highly abundant in the supernatants of PBMCs stimulated with iRBCs during the primary stimulation and the 3-day rest period, while it remained nearly undetectable in the RPMI and uRBC conditions (Figure 7A). For Hz, a subset of the healthy volunteers showed high IFN-γ levels during the primary stimulation, while a large portion did not have detectable levels of IFN-γ (Figure 7A). However, in the 3-day rest supernatants from samples stimulated with Hz, all healthy volunteers demonstrated elevated levels of IFN-γ relative to the RPMI and uRBC control conditions (Figure 7A). To determine what cells were producing IFN-γ within the first 12 hours of primary stimulation, we utilized a cytometry by time of flight panel to identify 9 cell populations in PBMCs (Supplemental Figure 5) and found that very few cells were producing IFN-γ in the RPMI and uRBC conditions, while in the iRBC condition, the total number of IFN-γ–producing cells increased dramatically (Figure 7B). The main cells producing IFN-γ were CD4^+^ and CD8^+^ T cells, NKT cells, NK cells, and monocytes (Figure 7B), indicating that together T lymphocytes, NKT cells, and NK cells make up the majority of IFN-γ–producing cells. In a separate set of experiments, we also found that γΔ T cells represented a small fraction of the IFN-γ–producing cells (Supplemental Figure 7). To test whether IFN-γ could restore hyperresponsiveness in purified monocytes, we stimulated monocytes with control stimuli, iRBCs, or Hz in the absence or presence of IFN-γ during the 24-hour primary stimulus and the 3-day rest period. We found that the addition of IFN-γ alone was sufficient to induce hyperproduction of TNF-α and IL-6 in purified monocytes (Figure 7, C and D), indicating that the induction of IFN-γ by iRBC or Hz is an important component of generating trained immune responses in monocytes. To test whether inhibition of IFN-γ receptor signaling could block trained immune responses, we treated PBMCs with the JAK/STAT inhibitor tofacitinib (29). Tofacitinib treatment at 2 μM blocked the production of CXCL10/IP-10 in PBMCs stimulated with iRBCs, Hz, or IFN-γ, indicating that IFN-γ receptor signaling was sufficiently inhibited (Supplemental Figure 6). PBMCs treated with 2 μM or 5 μM of tofacitinib had the same viability as PBMCs treated with DMSO solvent alone (Supplemental Figure 7). PBMCs stimulated with iRBCs, Hz, or IFN-γ were hyperresponsive to LPS or Pam_3_CSK_4_ restimulation in the presence of the DMSO solvent (Figure 7, E and F), whereas PBMCs treated with 2 μM of tofacitinib during the primary stimulus and 3-day rest failed to mount robust TNF-α or IL-6 responses upon restimulation (Figure 7, G and H). Altogether, these data showed that IFN-γ production was induced by *P*. *falciparum* iRBCs and Hz in PBMCs, and that IFN-γ signaling was critical to the development of trained innate immune responses to *P*. *falciparum*.

### Increased chromatin accessibility in child patients with malaria.

**Figure 8:**
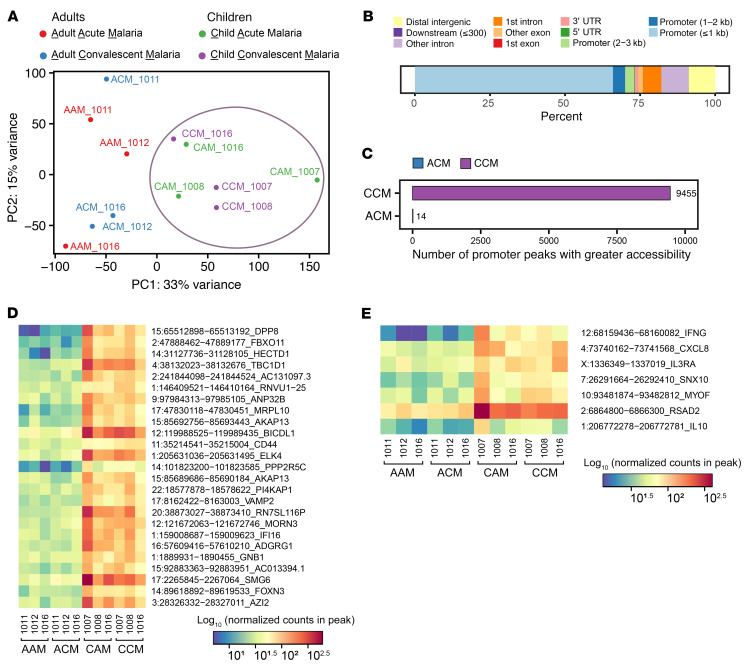
ATAC-Seq of monocytes from adults and children infected with malaria: increased chromatin accessibility in convalescent child patients. ATAC-Seq of monocytes from adult (*n =* 3) and child (*n =* 3) patients with malaria during acute disease and convalescence (after antimalarial treatment). (**A**) Principal component analysis of ATAC-Seq regularized log-transformed counts for each patient. The purple circle indicates the clustering of pediatric patients. (**B**) Genomic location of significant (adjusted *P* < 0.05) differentially accessible peaks between convalescent adult patients with malaria (ACM) and convalescent child patients with malaria (CCM). (**C**) The number of significant differentially accessible promoter peaks from **B**. (**D**) Heatmap of log_10_ normalized counts for the 25 most significant differentially accessible promoter peaks from **C**. (**E**) Heatmap of log_10_ normalized counts for 7 significant differentially accessible promoter peaks located at genes highlighted in the preceding RNA-Seq experiment (with red labels) as well as IL-10.

Part of the mechanism underlying hyperinflammatory responses in innate immune training is contingent upon chromatin modifications, which open the chromatin of genes, like TNF-α and IL-6, and allow for more robust expression of these genes (15, 30). Furthermore, inhibition of these chromatin modifications by histone methyltransferase inhibitors abrogates trained immune responses (13, 15). In Kisumu, Kenya, where malaria transmission is endemic, people are infected repeatedly with malaria over their lifespan. Although the first rounds of infection are marked by a highly febrile inflammatory disease (largely in children), over time, a milder or tolerogenic response to infection develops by the time individuals reach adulthood (31). We posit that the child patients with malaria represent a population that undergoes innate immune training, and the adult patients represent a population that undergoes tolerance. We thus hypothesized that pediatric patients with acute febrile malaria from Kisumu would, therefore, exhibit more open chromatin compared with adults at gene loci identified in our RNA-Seq to be most differentially expressed in iRBC-trained PBMCs (Figure 2). We collected PBMCs from 3 adults and 3 children during acute malaria infection, and then collected PBMCs from those same patients during convalescence after antimalarial treatment (28 weeks). We purified monocytes from these samples and performed assay for transposase-accessible chromatin with high-throughput sequencing (ATAC-Seq) to measure the chromatin accessibility across the genome. We performed a principal component analysis of ATAC-Seq data, which demonstrated that children had chromatin signatures that were distinct from adults during both acute and convalescent malaria (Figure 8A). As expected, the majority of significant differentially accessible peaks between adult convalescent patients with malaria and child convalescent patients were located in the promoter regions (Figure 8B). Among these differentially accessible chromatin peaks, the vast majority were in the children (Figure 8C), indicating that convalescent child patients with malaria had more accessible chromatin than the adult convalescent patients. Consistent with this, the top 25 differentially accessible chromatin regions were also more accessible in convalescent child patients with malaria than in adult convalescent patients (Figure 8D). The majority of these top 25 genes encode transcription factors or proteins involved in metabolism. Among the top 50 genes identified in our RNA-Seq experiment (Figure 2C), 5 of them (IFNG, CXCL8, IL3RA, SNX10, MYOF, and RSAD2) had promoter regions that were significantly more accessible in convalescent child patients with malaria than in adult convalescent patients (Figure 8E). Interestingly, the promoter region for IL-10 was also significantly more accessible in convalescent child patients with malaria than adults at the corresponding treatment stage.

## Discussion

The concept of innate immune training originated from early twentieth century observations that children who were vaccinated with Bacille Calmette-Guerin (BCG) had a remarkably reduced risk of death during the first few years of life compared with those who were unvaccinated. Surprisingly, it was clear that the protective effects of BCG were not due to acquired immunity to tuberculosis (TB) given that the decline in death rate was too large to be accounted for by TB, and most of the effect was seen in the first year of life, whereas TB was mostly fatal in older children. The investigators concluded that the “BCG vaccine provokes a nonspecific immunity” (32). A similar effect has been observed with other vaccines, including those against measles and diphtheria, tetanus, and pertussis (33).

Research since then has cast some light on these “nonspecific immunity” phenomena. As the effects of BCG appeared to occur independently of a specific acquired immune response, including the generation of robust T cell–mediated immunity or an antibody-induced effect, investigators focused on innate immunity and the concept that the innate immune system could develop an enhanced response (i.e., that it could undergo training). Indeed, this appeared to be the case, and this training could be examined in cell culture systems and in vivo animal models. Multiple studies have now shown that innate immune cells exposed to BCG or other microbial stimuli in culture have an enhanced ability to produce proinflammatory molecules when subsequently exposed to LPS or other TLR ligands (30). This training effect has been demonstrated in 2 major innate immune cell types, namely monocytes and NK cells, and was assumed to be a cell-intrinsic effect. The bulk of the evidence suggests that innate immune training is mediated by epigenetic and cellular metabolic alterations, which maintain this hyperinflammatory state.

It was with this background in mind that we posed the question of whether innate immune training occurred during malaria, a disease that affects billions of individuals over their lifetime. Our studies were initially intended to focus on monocytes, as previous studies had used the adherent fractions of PBMCs (which were assumed to be greatly enriched in phagocytes). Indeed, the premise of this experimental design was flawed, as our data showed, and hence an important role for lymphocyte populations in the training phenomena was missed. Nevertheless, the data reported here do indeed suggest that training is principally the job of phagocytes, as an analysis of the source of cytokines during training clearly demonstrated that the principal mediators of inflammation were monocyte- and DC-derived. Indeed, we believe that this training stimulus induced a subset of the monocytes to differentiate into DCs. Somewhat more surprising was that these cell populations existed in greater numbers because of the training process. This finding was not due to a cell expansion as we did not see evidence of increased proliferation of these cells; therefore, it is likely due to an increased ability of these cells to survive. Future studies into the precise survival mechanism will be important.

Although in vitro systems to evaluate trained immune responses to malaria are useful for controlled mechanistic studies, they are limited in their ability to fully model a living mammalian organism undergoing malaria infection. Primary human immune cells have limited longevity in cell culture and lack the continuous repopulation from the bone marrow that is present in vivo. For this reason, we looked at patients with malaria infection for evidence of a trained immune response by measuring their chromatin accessibility, a mechanism that is synonymous with trained immunity. The ATAC-Seq results revealed more accessible chromatin in the child cohort than the adult cohort. This aligns with the clinical course of disease in these 2 populations, namely that children with their first exposures to malaria exhibit symptoms of febrile malaria, whereas adults with a lifetime history of reinfection exhibit little or no symptoms of malaria disease when infected. Since our in vitro system used PBMCs from malaria-naive individuals, it modeled the first infection with malaria, similar to the child patients with malaria in this study. These findings correlate well with another study of patients with malaria in Mali, where the authors found that monocytes from children with no or limited malaria infection history had a more inflammatory response to malaria in in vitro stimulation, whereas monocytes from adults with a history of recurrent malaria infections over their lifespan exhibited a tolerant response (34).

As with any immune response to a pathogen that causes significant human disease, it remains to be defined how innate immune training might add to host defenses against malaria or, conversely, result in increased morbidity and mortality. Epidemiological observations have now clearly established that children with malaria are prone to potentially fatal bacterial infections. Most prominently, several studies have shown that children with malaria are more likely to have bacteremia, especially with non-*typhi Salmonella* infection (35, 36). The reasons for this susceptibility are unclear, but bacterial translocation (where bacteria or endotoxin breach the gut epithelial barrier) as a secondary consequence of infection is common in the clinic and can be reproduced in laboratory animals. On the one hand, innate immune training might be limiting bacterial invasion, and one might imagine that without enhanced cytokine responses, non-*typhi Salmonella* infections might be far more frequent. Alternatively, enhanced cytokine production as a result of bacterial infection might be a cause of sepsis accompanied by organ dysfunction and failure, which in turn might result in enhanced bacterial invasion.

Indeed, one can imagine that the local innate immune response to sequestered infected erythrocytes in the venules of the CNS and elsewhere might be central to the development of increased brain volume, herniation, and death in cerebral malaria. Under this scenario, it is hypothesized that cerebral malaria, in its worst forms, might be driven by bacterial translocation (or the translocation of bacterial products, such as lipopeptides and LPS from the gut into the body), resulting in a cytokine storm. All of this is quite far from the usual concepts of trained immunity that are derived from the long-term observations of vaccines. Unlike the BCG or measles vaccine, both of which are live vaccines that have benefits years later unrelated to their primary purpose, there is no such observation of enhanced survival in individuals who have malaria. This could be due either to a lack of epidemiologic data, which is unlikely, or to a fundamental difference in the biology of a live pathogenic infection and the low-grade infection induced by an attenuated organism. The analysis of patients with malaria is further complicated because these patients almost universally experience severe poverty and are often reinfected multiple times throughout their lives. These multiple infections and the epigenetic changes associated with them (as demonstrated by the apparent changes in chromatin accessibility suggested in Figure 8) might partially explain the inability of individuals in malaria-endemic regions to develop long-lasting immunity to our best malaria vaccines. For example, both the circumsporozoite-based vaccine RTS,S/AS01 or a vaccine consisting of large numbers of live irradiated sporozoites are more effective at inducing an immune response in malaria-naive individuals than in individuals from endemic areas (37, 38).

Finally, this work again points to a central role for IFNs in the innate immune response to malaria. These in vitro studies clearly demonstrated that a soluble factor or factors from lymphocytes were necessary to observe training. This is a similar mechanism to another report where alveolar macrophages during viral infection require T lymphocyte IFN-γ for the initial priming phase but maintain autonomous reprogramming as memory alveolar macrophages after the fact (28). In the in vitro system, the mechanism for activation of T cell–derived IFN-γ is most likely a classical antigen presentation scenario where the monocytes take up and present malarial antigen to T cells, activating them and inducing their production of IFN-γ and other lymphokines. The Transwell experiments introduced the question of whether the monocyte-elicited activation of T cells to produce soluble factors like IFN-γ may be happening in the absence of direct cell-cell antigen presentation; however, we cannot eliminate the possibility that antigen presentation occurs in this system via the release of exosomes containing peptide:MHC that can pass through the Transwell pores. Indeed, understanding the cellular crosstalk between monocytes and lymphocytes will be an important area of study. While it seems that the phenomena of monocyte training cannot entirely be due to an IFN-γ response, the inhibition of IFN-γ and, conversely, the addition of IFN-γ to the culture medium to reproduce training suggested that IFN-γ is a major cytokine responsible for lymphocyte-monocyte communication. Any efforts to improve the adverse outcomes in severe malaria must take into account the complex role of IFN-γ, both in terms of its protective effect against overwhelming parasitemia and the end organ damage that it might mediate.

## Methods

### Study population.

Venous blood was collected in sterile 60 mL syringes (BD Biosciences) pretreated with heparin (Sagent Pharmaceuticals) and a BD Vacutainer 21G push button blood collection set butterfly needle (BD Biosciences). Donors for in vitro experiments at University of Massachusetts Chan Medical School were all malaria-naive. The donor age range was 25 to 65 years, with a median age of 35 years. Donor sex distribution was 33.3% female and 66.6% male.

### Malaria cultures and IRBC/Hz isolation.

The *P*. *falciparum* strain 3D7 was grown in vitro in malaria media consisting of RPMI 1640 powder medium (Gibco, Thermo Fisher Scientific) and cell culture–grade water (Corning) supplemented with HEPES (Thermo Fisher Scientific), hypoxanthine (MilliporeSigma), AlbuMAX II (Gibco, Thermo Fisher Scientific), sodium bicarbonate (Gibco, Thermo Fisher Scientific), and gentamicin reagent solution (Gibco, Thermo Fisher Scientific), with purified human erythrocytes 21 days old or less at a hematocrit of 5%. Purified iRBCs were isolated by passing 3D7-infected erythrocyte cultures over Miltenyi Biotec LD magnetic columns on a QuadroMACS Separator magnet, washed, and eluted with sterile PBS. Cells were counted on a hemacytometer for total cell number, and a slide of 10 μL of iRBCs was stained with Giemsa stain (MilliporeSigma) to enumerate the percentage of parasitemia. Purified iRBC cultures had 90% or greater parasitemia. Hz was isolated from spent media from 3D7 malaria cultures by passing through magnetic LS columns in a QuadroMACS magnet (Miltenyi Biotec) and eluting in sterile water. The Hz concentration was measured by solubilizing in 20 mM NaOH for 2 hours and then using the QuantiChrom Heme Assay kit (BioAssay Systems), according to the manufacturer’s instructions. Hz stocks were stored at –20°C.

### Cell isolations.

Human erythrocytes were purified from whole blood from healthy donors by centrifuging at 1000*g* for 7 minutes at 25°C with low acceleration and low deceleration. The serum and buffy coat layers were removed, and the erythrocytes were washed by resuspending in malaria media, centrifuging at 1000*g* for 7 minutes at 4°C with low acceleration and low deceleration, and removal of malaria media. The erythrocytes were washed a total of 3 times and then resuspended 1:2 in malaria media for a hematocrit of 50%. Purified erythrocytes were stored at 4°C for 2 to 3 weeks. For PBMC isolation, whole blood from healthy donors was diluted 1:2 in sterile PBS (Corning) and overlaid on Ficoll Paque-PLUS (GE Healthcare) in 50 mL conical tubes and centrifuged at 800*g* for 15 minutes at 25°C with no brake. The resulting buffy coats were collected and washed twice in RPMI 1640 medium (Corning) supplemented with L-glutamine (Gibco, Thermo Fisher Scientific), sodium pyruvate (MilliporeSigma), and gentamicin reagent solution (Gibco, Thermo Fisher Scientific), and then cells were treated with RBC lysis buffer (Roche) for 3 minutes at room temperature. Cells were washed in RPMI and counted on a hemacytometer. PBMCs were plated at 5 × 10^5^ cells per well in 96-well round-bottom plates (Falcon) or 5 × 10^6^ cells per well in 6-well flat-bottom plates (Falcon) and incubated at 37°C for 1 hour. Nonadherent cells were removed by washing 3× with sterile PBS. All cell isolations using Miltenyi Biotec kits were performed according to the manufacturer’s instructions, with the exception of the MACS buffer, which was substituted with sterile PBS supplemented with 2% FBS (Atlanta Biologicals) and then filtered over a 0.22 μM filter. Purified monocytes were obtained from PBMCs using the Pan Monocyte Isolation kit (Miltenyi Biotec). Cell purity was confirmed by flow cytometry, and the resulting monocytes had a purity of 95% or more. PBMCs were depleted of CD3^+^ cells using the human CD3 Microbeads kit (Miltenyi Biotec) over an LD column (Miltenyi Biotec), with 0.2% or less of CD3^+^ cells remaining after depletion. PBMCs were depleted of CD56^+^ cells using the human CD56 Microbeads kit (Miltenyi Biotec) over an LD column, with 0.9% or less of CD56^+^ cells remaining after depletion. For all cell purifications, a sham isolation was run on PBMCs by resuspending cells in MACS buffer and running them over an LD column in a QuadroMACS magnet (Miltenyi Biotec). For the Transwell assays, PBMCs were depleted of CD14^+^ cells using the Human CD14 Microbeads kit (Miltenyi Biotec) over an LD column, with 0.5% or less of CD14^+^ cells remaining after depletion. Purified CD4^+^ T cells were isolated from PBMCs using the human CD4^+^ T cell Isolation kit (Miltenyi Biotec) over an LD column, and the resulting CD4^+^ T cells had a purity of 97% or more.

### Cell stimulations.

Adherent PBMCs were incubated in RPMI 1640 GlutaMAX (Gibco, Thermo Fisher Scientific) supplemented with 10% human male AB serum (MilliporeSigma) and gentamicin reagent solution (Gibco, Thermo Fisher Scientific) and stimulated for 24 hours with culture medium, 1 × 10^6^ uRBCs, 1 × 10^6^ iRBCs, or 50 μM Hz unless otherwise specified. After primary stimulation, cells were washed once with PBS and allowed to rest in RPMI for 3 days. Cells were then stimulated with 10 ng/mL ultrapure *E*. *coli* 0111/B4 LPS (InvivoGen) or 10 μg/mL Pam_3_CSK_4_ (InvivoGen) for 4 to 24 hours. Stimulated cells were harvested for RNA or cytometry analysis, and supernatants were frozen at –20°C for subsequent cytokine measurement. For experiments with IFN-γ stimulation, 20 ng/mL of human IFN-γ (PeproTech) final concentration was added to cells during the primary 24-hour stimulation and during the 3-day rest period. For experiments with JAK/STAT inhibition, tofacitinib (Selleck Chemicals) was added to the medium at a final concentration of 1 μM, 2 μM, or 5 μM, and cells were incubated with tofacitinib for 1 to 2 hours before adding primary stimuli. As a solvent control, DMSO was diluted in the same manner as tofacitinib and added to cells. Tofacitinib or DMSO was also added to the media during the 3-day rest period. Sham-sorted PBMCs, CD3-depleted PBMCs, and CD56-depleted PBMCs were plated at the same cell density as adherent PBMCs. Purified monocytes were plated at 1 × 10^5^ cells per well in 96-well round-bottom plates (Falcon). For Transwell experiments, monocytes were plated at 4 × 10^5^ cells per well in 12-well or 24-well plates and stimulated with RPMI, 5.8 × 10^6^ uRBCs, or 5.8 × 10^6^ iRBCs. In a Transwell with 0.4 μm pores (Falcon), either 2.5 × 10^6^ CD14-depleted PBMCs or 2 × 10^6^ purified CD4^+^ T cells were plated.

### Cytokine measurement.

Cell supernatants were thawed and diluted in reagent diluent to measure cytokines. TNF-α, IL-6, IP-10/CXCL10, and IFN-γ Duo-Set ELISA kits were from R&D Systems and performed according to the manufacturer’s instructions. Absorbance was read on a SpectraMax iD5 machine (Molecular Devices) at 450 nm with 570 nm subtraction.

### mRNA isolation and RNA-Seq library preparation.

PBMCs were isolated from 3 individuals using the methods described above. Cells from these biological replicates were exposed to iRBCs, uRBCs, or RPMI for 24 hours and rested 3 days prior to stimulation with PAM_3_CSK_4_ (10 μg/mL) for 0, 4, and 12 hours. Total RNA was extracted using the Aurum Total RNA Mini kit (Bio-Rad). Samples were eluted in nuclease-free water and analyzed for concentration and fragment analysis by the Molecular Biology Core Lab at University of Massachusetts Chan Medical School using an Agilent Fragment Analyzer. For each sample, 0.5 μg of total RNA was used to prepare RNA-Seq libraries using the TruSeq Stranded Total RNA Library Prep Human/Mouse/Rat kit and the TruSeq RNA single indexes set A (Illumina) according to the manufacturer’s instructions. Samples were sequenced at the Bauer Sequencing Core at Harvard University on a HiSeq 2500 (Illumina), using paired-end 75 bp sequencing.

### Flow cytometry and mass cytometry.

For surface phenotyping, cells were washed in FACS buffer consisting of PBS and 2% FBS, centrifuged at 450*g* and resuspended in 100 μL of surface antibodies diluted 1:20 in FACS buffer. Samples were stained for 30 minutes at 4°C in the dark and then washed twice with an excess of FACS buffer. Samples were then fixed in Affymetrix 4% paraformaldehyde (Thermo Fisher Scientific) for 20 minutes at 4°C in the dark, centrifuged at 800*g* for 5 minutes, and resuspended in FACS buffer for acquisition. For experiments to determine cell purity after purification, samples were acquired on a BD Biosciences LSR flow cytometer equipped with blue 488 nm, violet 407 nm, ultraviolet 355 nm, and red 633 nm lasers. For intracellular cytokine analysis after LPS and Pam_3_CSK_4_ stimulation, cells were incubated for 5 hours in medium or TLR agonists along with brefeldin A (eBioscience, Thermo Fisher Scientific). After stimulation, media was removed and cells were resuspended in 2 mM EDTA in PBS and incubated for 15 minutes at 37°C. Cells were then surface-stained as described above with the addition of ZombieGreen Viability dye (BioLegend) diluted 1:400, followed by fixation and permeabilization with BD Biosciences Cytofix/Cytoperm for 20 minutes at 4°C in the dark. Cells were washed with 1× perm wash buffer (BD Biosciences) and resuspended in 100 μL cytokine antibodies diluted 1:20 in 1× perm wash buffer and incubated for 30 minutes at 4°C in the dark. Cells were washed twice with an excess of perm wash buffer and resuspended in FACS buffer for analysis on a Cytek Biosciences Aurora cytometer equipped with violet, blue, yellow/green, and red lasers and SpectroFlo software. For intracellular cytokine analysis during primary stimulation with *P*. *falciparum–*infected erythrocytes, cells were incubated for 12 hours with corresponding stimuli, and brefeldin A was added for the final 4.5 hours of stimulation. Cells were harvested as described above, washed in PBS, and resuspended in 5 μM solution of Cell-ID Cisplatin (Fluidigm) for 10 minutes at room temperature. Cells were washed twice in an excess of Maxpar Cell Staining buffer (Fluidigm), resuspended in a 50 μL solution of human TruStain FcX (BioLegend) Fc Receptor block diluted 1:20 in Maxpar Cell Staining buffer, and incubated for 15 minutes at 4°C. After incubation, surface-staining antibodies in 50 μL of Maxpar Cell Staining buffer were added, and cells were incubated for 30 minutes at room temperature. Cells were washed twice with an excess of Maxpar Cell Staining buffer, resuspended in 50 μL of anti-APC 176Yb (Fluidigm) diluted 1:100 in Maxpar Cell Staining buffer, and incubated for 15 minutes at room temperature. Cells were washed twice with an excess of Maxpar Cell Staining buffer, resuspended in 1 mL of Maxpar Fix and Perm buffer (Fluidigm), and incubated for 30 minutes at room temperature. Cells were spun at 800*g* for 5 minutes at 4°C, resuspended in 50 μL of intracellular cytokine antibodies diluted in Maxpar Perm-S buffer (Fluidigm), and incubated for 30 minutes at room temperature in the dark. Cells were then washed twice with an excess of Maxpar Cell Staining buffer, resuspended in 1 mL of Cell-ID Intercalator Ir (Fluidigm) diluted 1:1000 in Maxpar Fix and Perm buffer, and incubated overnight at 4°C. Cells were washed twice with an excess of Maxpar Cell Staining buffer, and then washed twice with an excess of Maxpar water (Fluidigm). Cells were resuspended in Maxpar water and a 1:10 dilution of EQ Four Element calibration beads (Fluidigm) and run on a Helios CyTOF system (Fluidigm). For a complete list of all antibodies used, please see Supplemental Table 1.

### Graphics.

Bar graphs were generated using GraphPad Prism version 8. tSNE plots were generated using FlowJo version 10.6.0 for Mac (BD).

### Analysis of RNA-Seq data.

We used Bowtie2 (39) to align RNA-Seq reads to the human genome (assembly GRCh38, Ensembl version 84). The RSEM package (40) was used to calculate transcripts per million (TPM) mapped reads for each sample. We used the EBSeq-HMM package (41) to identify genes that were differentially expressed over a given time-course experiment. Here, we controlled for false discovery at a rate of 5%, which corresponds to a posterior probability of 0.05 or less for a constant path of expression along the time course. We calculated fold change values between the main conditions (uRBCs and iRBCs) at each timepoint. We rank-ordered all genes that were differentially expressed in either of the main time courses (uRBCs and iRBCs) by their maximum absolute fold change values. For GO enrichment analysis (42), the top 100 ranked genes (all genes were a minimum of 3-fold) were analyzed. Custom plots were generated within the R software environment (43) and the ggplot2 package (44). The RNA-Seq data and associated protocols are available from the ArrayExpress public repository (E-MTAB-9066).

### Flow cytometry and CyTOF analysis.

All flow cytometry and CyTOF fcs files were analyzed in FlowJo version 10.6.0 for Mac (Becton Dickinson). For the Cytek Aurora flow cytometer, samples were compensated and autofluorescence was subtracted out in the SpectroFlo software to generate “unmixed”.fcs files. Both raw and unmixed.fcs files were exported, but only unmixed.fcs files were uploaded into FlowJo for analysis. Fluorescence-minus-one (FMO) controls were run for ZombieGreen, CD14-APC, CD11c-FITC, CD3-PE, and CD56-BV605, and these FMO samples were used to determine appropriate gating. For tSNE analysis in FlowJo, samples were first concatenated together and then down-sampled to 90,000 events, and then tSNE was calculated using the following parameters: opt-SNE with KNN algorithm-exact (vantage point tree) and Barnes-Hunt gradient algorithm with perplexity 30, Eta (learning rate) 6300, and iterations 1000.

### Monocyte isolation from samples from patients with malaria.

Frozen samples of PBMCs from Kenyan adults and children with acute *P*. *falciparum* malaria, as well as matched convalescent (*P*. *falciparum* PCR negative) samples acquired 28 days after treatment, were provided by Case Western Reserve. Monocytes were isolated from PBMCs using Pan Monocyte Isolation kit (Miltenyi Biotec, 130-096-537) following the manufacturer’s instructions.

### ATAC-Seq of monocytes from patients with malaria.

ATAC-Seq on purified monocytes was performed as described previously by Corces et al. without modifications (45). A preamplification of transposed nuclear fragments was performed using primers with Illumina adaptors. The reaction mixture consisted of 2.5 μL 25 μM primers, 25 μL 2× NEBNext Master Mix (New England Biolabs, M0541S), and 20 μL of the transposed sample for preamplification PCR (72°C × 5 minutes, 98°C × 30 seconds, 5 cycles of 98°C × 10 seconds, 63°C × 30 seconds, 72°C × 1 minute, hold at 4°C). The optimal cycle number for analysis was determined by qPCR using 5 μL of the preamplified product to define the necessary additional numbers of cycles as described by Buenrostro et al. (46), and the library was amplified accordingly. The Zymo DNA Clean and Concentrator-5 kit was used for purification, and fragment size distribution was assessed with the High Sensitivity DNA ScreenTape assay D1000 kit (Agilent Technologies, 5067-5584). DNA concentration was determined by Qubit fluorometer (Life Technologies). High-throughput DNA sequencing of the tagmented library was performed on a NextSeq 500 System (Illumina) to generate paired-end 75 bp reads using a NextSeq 500/550 High-Output v2.5 kit (Illumina, 20024906).

### Analysis of ATAC-Seq data.

We used the Cutadapt program (47) with options -a CTGTCTCTTATACACATCT -A AGATGTGTATAAGAGACAG -m 20 and --nextseq-trim 20 to remove the respective adapter sequences. We used BWA-MEM (48) with the -M option to align paired-end ATAC-Seq reads to the human genome (assembly GRCh38, Ensembl version 100). We used the PICARD MarkDuplicates program with the “Remove duplicates true” option to remove duplicates. We used the MACS2 callpeak program (49) with --format BAMPE -g 2.7e9 -B --*q*value 0.05 options to determine peaks. To determine differentially accessible peaks, we used the DESeq2 package (50) to compare narrowPeak data between adult convalescent patients and child convalescent patients. A peak was deemed significant when the adjusted *P* value was less than 0.05. The ChIPseeker package (51) was used to annotate and plot the genomic regions for each peak. Custom plots were generated within the R software environment (43) and the ggplot2 package (44). The ATAC-Seq data and associated protocols are available from the ArrayExpress public repository (E-MTAB-11643).

### Materials availability.

This study did not generate new, unique reagents.

### Statistics.

Statistics were computed in GraphPad Prism version 8. Data were first subjected to a normality test; a majority of the data were non-normally distributed, so we performed nonparametric statistical tests for all the analyses. A Kruskal-Wallis test was used to determine 2-way ANOVA, and a Dunn’s multiple-comparison test was run to determine whether there was a significant difference between individual groups (e.g., iRBCs, Hz) and the uRBC control group. For the monocyte training with and without IFN-γ experiment, a Kruskal-Wallis test was used to determine 2-way ANOVA and a Dunn’s multiple-comparison test to determine whether there was a significant difference between individual treatment groups with and without IFN-γ treatment (e.g., RPMI vs. RPMI + IFN-γ) was used. *P* ≤ 0.05 was considered significant.

### Study approval.

The human studies were approved by the University of Massachusetts Chan Medical School IRB (protocols 12497 and 10368). Ethical approval was obtained from the IRB of University Hospitals Cleveland Medical Center (IRB protocol 06-11-22 children, IRB protocol 12-16-23 adults), and the Kenya Medical Research Institute Ethical Review Committee (SSC 2207 children, SSC 3455 adults). All donors provided written informed consent prior to the blood draw.

## Author contributions

JNC conceptualized the study. JNC, DRC, LDSS, and KD contributed to methodology. DRC contributed software. JNC, DRC, and LDSS conducted the investigation. JNC, DRC, and EAKJ performed visualization. JNC and DRC conducted formal analysis. JNC, DTG, and DRC wrote the original draft. JNC, DRC, EAKJ, KAF, and DTG contributed to review and editing of the manuscript. KAF, DTG, AD, and JNC acquired funding. KAF, DTG, and AD supervised the study.

## Supplementary Material

Supplemental data

## Version 1 06/01/2022

Electronic publication
