# Supplementary material for: Lymphocyte crosstalk is required for monocyte-intrinsic trained immunity to Plasmodium falciparum
Source: J Clin Invest. 2022 Jun 1;132(11):e139298. doi: 10.1172/JCI139298 (PMC9151696; doi:10.1172/JCI139298)
Supplement: Supplemental data [file jci-132-139298-s059.pdf]

Key Resources Table

REAGENT or RESOURCE	SOURCE	IDENTIFIER
Antibodies		
Anti-human CD3 (UCHT1) 170Er	Fluidigm	3170001B;<br>RRID: AB_2661807
Anti-human CD4 (RPA-T4) 145Nd	Fluidigm	3145001B;<br>RRID: AB_2661789
Anti-human CD8 (SK1) 168Er	Fluidigm	3168002B
Anti-Human CD45RA (HI100) 155Gd	Fluidigm	3155011B;<br>RRID: AB_2810246
Anti-human CD19 (HIB19) 142Nd	Fluidigm	3142001B;<br>RRID: AB_2651155
Anti-human HLA-DR (L243) 174Yb	Fluidigm	3174001B;<br>RRID: AB_2665397
Anti-human CD14 (RMO52) 148Nd	Fluidigm	3148010B
anti-human CD16 (3G8) 209Bi	Fluidigm	3209002B;<br>RRID: AB_2756431
anti-human CD11c (Bu15) 147Sm	Fluidigm	3147008B;<br>RRID: AB_2687850
anti-human CD56 (NCAM16.2) 163Dy	Fluidigm	3163007B
anti-human CD123/IL-3R (6H6) 143Nd	Fluidigm	3143014B;<br>RRID: AB_2811081
anti-human CD303/BDCA2 (201A) 153Eu	Fluidigm	3153007B
anti-human CD141 (1A4) 173Yb	Fluidigm	3173002B;<br>RRID: AB_2714156
anti-human IL-10 (JES3-9D7) 166Er	Fluidigm	3166008B
anti-human IL-6 (MQ2-13AS) 156Gd	Fluidigm	3156011B;<br>RRID: AB_2810973
anti-human IFNg (B27) 165Ho	Fluidigm	3165002B
anti-human TNFa (Mab11) 152Sm	Fluidigm	3152002B
anti-human GM-CSF (BVD2-21C11) 159Tb	Fluidigm	3159008B
anti-human IL-2 (MQ1-17H12) 158Gd	Fluidigm	3158007B
anti-human CD38 (HIT2) 172Yb	Fluidigm	3172007B;<br>RRID: AB_2756288
anti-human CD86/B7.2 (IT2.2) 150Nd	Fluidigm	3150020B;<br>RRID: AB_2687852
anti-human CD274/PDL1 (29E.2A3) 175Lu	Fluidigm	3175017B
Anti-APC (APC003) 176Yb	Fluidigm	3176007B;<br>RRID: AB_2811236
APC anti-human CD1c antibody (L161)	BioLegend	331524;<br>RRID: AB_10718511
Human TruStain FcX (Fc Recepter Blocking Solution)	BioLegend	422302
APC/Cyanine7 anti-human CD1c (L161)	BioLegend	331519;<br>RRID: AB_10643413
Brilliant Violet 510 anti-human CD4 (OKT4)	BioLegend	317443;<br>RRID: AB_2561377
Brilliant Violet 605 anti-human CD56 (NCAM) (5.1H11)	BioLegend	362537;<br>RRID: AB_2565855
Brilliant Violet 650 anti-human CD8a (RPA-T8)	BioLegend	301042;<br>RRID: AB_2563505
Brilliant Violet 785 anti-human CD123 (6H6)	BioLegend	306031;<br>RRID: AB_2566448

Pacific Blue anti-human CD19 (SJ25C1)	BioLegend	363035;<br>RRID: AB_2632787
PE/Cy7 anti-human CD68 (Y1/82A)	BioLegend	333815;<br>RRID: AB_2562935
PerCP/Cy5.5 anti-human CD141 (Thrombomodulin) (M80)	BioLegend	344111;<br>RRID: AB_2561624
Anti-human CD16 PerCP-eF710	eBioscience	46-0168-42
APC anti-human CD14 (61D3)	Tonbo Biosciences	20-0149T100;<br>RRID: AB_2621560
FITC anti-human CD11c (3.9)	Tonbo Biosciences	35-0116-T100;<br>RRID: AB_2621678
redFluor 710 anti-human HLA-DR (L243)	Tonbo Biosciences	80-9952-T100;<br>RRID: AB_2622001
PE anti-human CD3 (Hit3a)	Tonbo Biosciences	50-0039-T100;<br>RRID: AB_2621735
BV750 Mouse anti-human TNF (MAb11)	BD Biosciences	566359;<br>RRID: AB_2739709
BV421 rat anti-human IL-6 (MQ2-13A5)	BD Biosciences	563279;<br>RRID: AB_2738113
Biological Samples		
Human male AB serum	Sigma	H4522-100ML
Fetal Bovine Serum	Atlanta Biologicals	S11150
Human PBMC from healthy donors	This Study	
Human erythrocytes from healthy donors	This Study	
Chemicals, Peptides, and Recombinant Proteins		
Human IFN gamma	Peprotech	300-02
Hemozoin purified from *P.f.* 3D7	This paper	
Tofacitinib (CP-690550, Tasocitinib)	Selleck Chemicals	S2789
PBS, 1X without calcium and magnesium	Corning	21-040-CV
RPMI Medium 1640 powder (+)L-Glutamine (-)Sodium Bicarbonate	Gibco	31800-022
RPMI 1640, 1X with L-glutamine	Corning	10-040-CV
L-Glutamine, USP	Gibco	21051-024
HEPES	Fisher	BP310-100
Sodium bicarbonate (7.5%)	Gibco	25080-094
Sodium Pyruvate	Sigma	P5280-25G
Gentamicin Reagent Solution	Gibco	15750-060
Red Blood Cell Lysis Buffer	Roche	11814389001
Giemsa Stain, Modified Solution	Sigma	48900-500ML-F
Albumax II	Gibco	11021-037
Ultrapure E. coli 0111:B4 LPS	Invivogen	tlrl-3pelps
Pam3CSK4	Invivogen	tlrl-pms
DMSO Hybri-Max	Sigma	D2650
Affymetrix 4% Paraformaldehyde	ThermoFisher	J19943-K2
eBioscience Brefeldin A	Invitrogen	00-4506-51
EDTA	Boston BioProducts	BM-150
Cell Culture Grade Water	Corning	25-055-CM
Heparin Sodium Injection, USP	Sagent Pharmaceuticals	NDC 25021-400-10

Hypoxanthine	Sigma	H9377-25G
Critical Commercial Assays		
Human IFN-gamma DuoSet ELISA	R&D Systems	DY285B-05
Human CXCL10/IP-10 DuoSet ELISA	R&D Systems	DY266-05
Human IL-6 DuoSet ELISA	R&D Systems	DY206
Human TNF-alpha DuoSet ELISA	R&D Systems	DY210
CD4+ T cell isolation kit, human	Miltenyi Biotec	130-096-533
Pan Monocyte isolation kit, human	Miltenyi Biotec	130-096-537
CD3 MicroBeads, human	Miltenyi Biotec	130-050-101
CD14 MicroBeads, human	Miltenyi Biotec	130-050-201
CD56 MicroBeads, human	Miltenyi Biotec	130-050-401
Aurum Total RNA Mini Kit	Bio-Rad	732-6820
TruSeq Stranded Total RNA library Prep Human/Mouse/Rat	Illumina	20020596
TruSeq Single Indexes Set A	Illumina	20020492
QuantiChrom Heme Assay kit	BioAssay Systems	DIHM-250
Deposited Data		
Experimental Models: Organisms/Strains		
Plasmodium falciparum strain 3D7	BEI Resources, MR4	MRA-102
Software and Algorithms		
Flowjo version 10	Becton, Dickinson and Co.	https://www.flowjo.com/ RRID: SCR_008520
Prism version 8	GraphPad	https://www.graphpad.com/scientific-software/prism/ RRID: SCR_002798
Illustrator, 23.1.1	Adobe	https://www.adobe.com/products/illustrator.html RRID: SCR_010279
Bowtie2	Langmead and Salzberg, 2012 PMID: 22388286	http://bowtie-bio.sourceforge.net/bowtie2/index.shtml
RSEM	Li and Dewey, 2011 PMID: 21816040	https://deweylab.github.io/RSEM/ RRID: SCR_013027
EBSeq-HMM	Leng et al. 2015 PMID: 25847007	http://bioconductor.org/packages/release/bioc/html/EBSeqHMM.html

Gene Ontology (GO)	Falcon and Gentleman, 2007<br>PMID: 17098774	http://geneontology.org/<br>RRID: SCR_002811
R	R Core Team, 2018	https://www.r-project.org/<br>RRID: SCR_001905
ggplot2	H. Wickham, 2009<br>ISBN: 978-0-387-98141-3	https://ggplot2.tidyverse.org/<br>RRID: SCR_014601
Other		
Maxpar Cell staining Buffer	Fluidigm	201068
Maxpar Fix and Perm Buffer	Fluidigm	201067
EQ Four Element Calibration beads	Fluidigm	201078
Cell-ID Cisplatin	Fluidigm	201064
Cell-ID Intercalator-Ir 125 uM	Fluidigm	201192A
Maxpar Perm-S Buffer	Fluidigm	201066
LS columns	Miltenyi Biotec	130-042-401
LD columns	Miltenyi Biotec	130-042-901
Ficoll Paque-PLUS	GE Healthcare	17144002
QuadroMACS Separator	Miltenyi Biotec	130-090-976
BD Cytofix/Cytoperm	BD Biosciences	554714
Zombie Green Dye	BioLegend	77476
OneComp eBeads	Invitrogen	2020-06-30

PCR Primers with Illumina adapters

Ad1_noMX	AATGATACGGCGACCACCGAGATCTACACTCGTCGGCAGCGTCAGATGTG
Ad2.1_TAAGGCGA	CAAGCAGAAGACGGCATACGAGATTCGCCTTAGTCTCGTGGGCTCGGAGATGT
Ad2.2_CGTACTAG	CAAGCAGAAGACGGCATACGAGATCTAGTACGGTCTCGTGGGCTCGGAGATGT
Ad2.3_AGGCAGAA	CAAGCAGAAGACGGCATACGAGATTTCTGCCTGTCTCGTGGGCTCGGAGATGT
Ad2.7_CTCTCTAC	CAAGCAGAAGACGGCATACGAGATGTAGAGAGGTCTCGTGGGCTCGGAGATGT
Ad2.8_CAGAGAGG	CAAGCAGAAGACGGCATACGAGATCCTCTCTGGTCTCGTGGGCTCGGAGATGT
Ad2.9_GCTACGCT	CAAGCAGAAGACGGCATACGAGATAGCGTAGCGTCTCGTGGGCTCGGAGATGT
Ad2.11_AAGAGGCA	CAAGCAGAAGACGGCATACGAGATTGCCTCTTGTCTCGTGGGCTCGGAGATGT
Ad2.16_CCGTTTGT	CAAGCAGAAGACGGCATACGAGATACAAACGGGTCTCGTGGGCTCGGAGATGT
Ad2.17_TGCTGGGT	CAAGCAGAAGACGGCATACGAGATACCCAGCAGTCTCGTGGGCTCGGAGATGT
Ad2.18_GAGGGGTT	CAAGCAGAAGACGGCATACGAGATAACCCCTCGTCTCGTGGGCTCGGAGATGT
Ad2.22_TGGTCACA	CAAGCAGAAGACGGCATACGAGATTGTGACCAGTCTCGTGGGCTCGGAGATGT
Ad2.24_CCACTCCT	CAAGCAGAAGACGGCATACGAGATAGGAGTGGGTCTCGTGGGCTCGGAGATGT

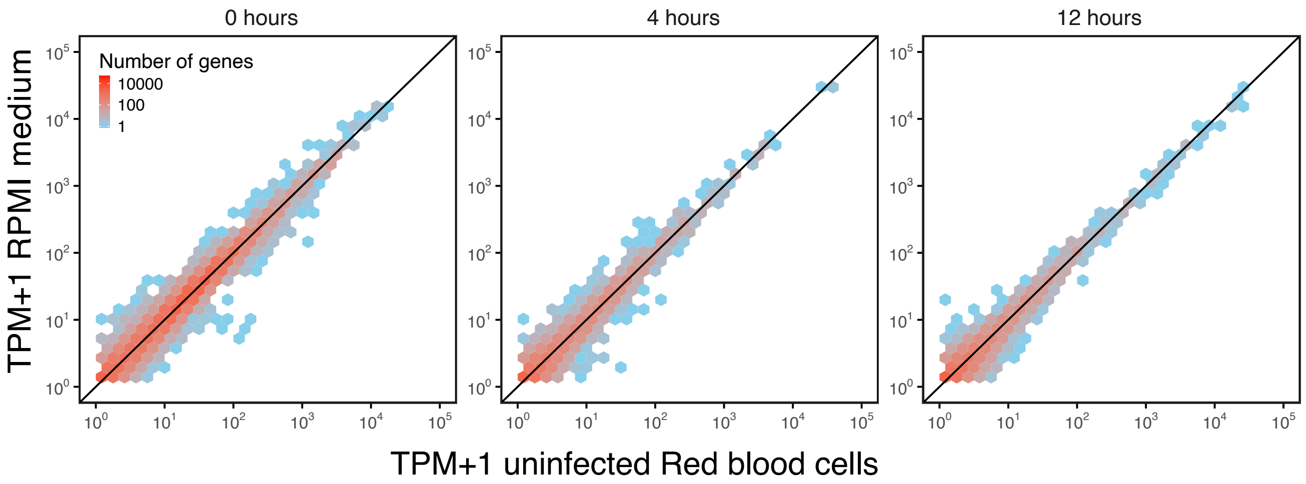
0 hours
4 hours
12 hours
Number of genes
10000
100
1
TPM+1 RPMI medium
TPM+1 uninfected Red blood cells

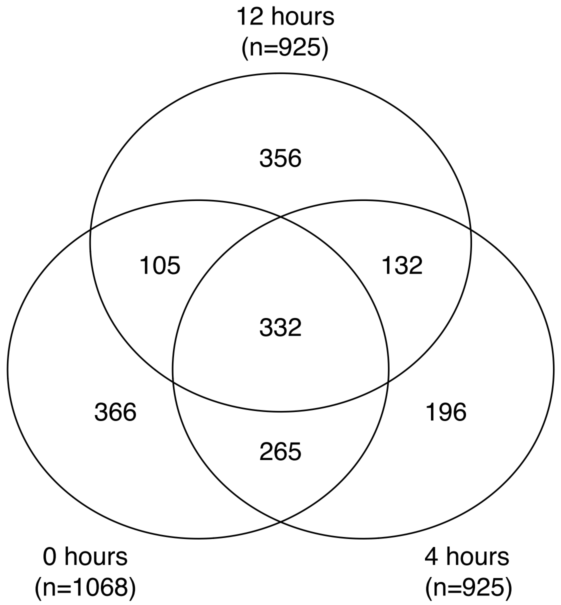
12 hours
(n=925)
356
105
132
332
366
196
265
0 hours
(n=1068)
4 hours
(n=925)

Supplementary Figure 1: RPMI control is similar to uRBC control and a summary of the differences between iRBC and uRBC. (A) RNA expression of PBMCs recently exposed to RPMI or uninfected RBCs and subsequently stimulated with PAM_3CSK_4 (after 0, 4, and 12 hours). RNA expression units correspond to Transcripts Per Million (TPM) mapped reads and are the average of three biological replicates. Each hexagonal bin shows the number of genes with the corresponding expression level. (B) Venn diagram showing the number of genes that are differentially expressed over either of the PAM_3CSK_4 time courses that also have a 3-fold or greater difference between uRBC and iRBC at the same times.

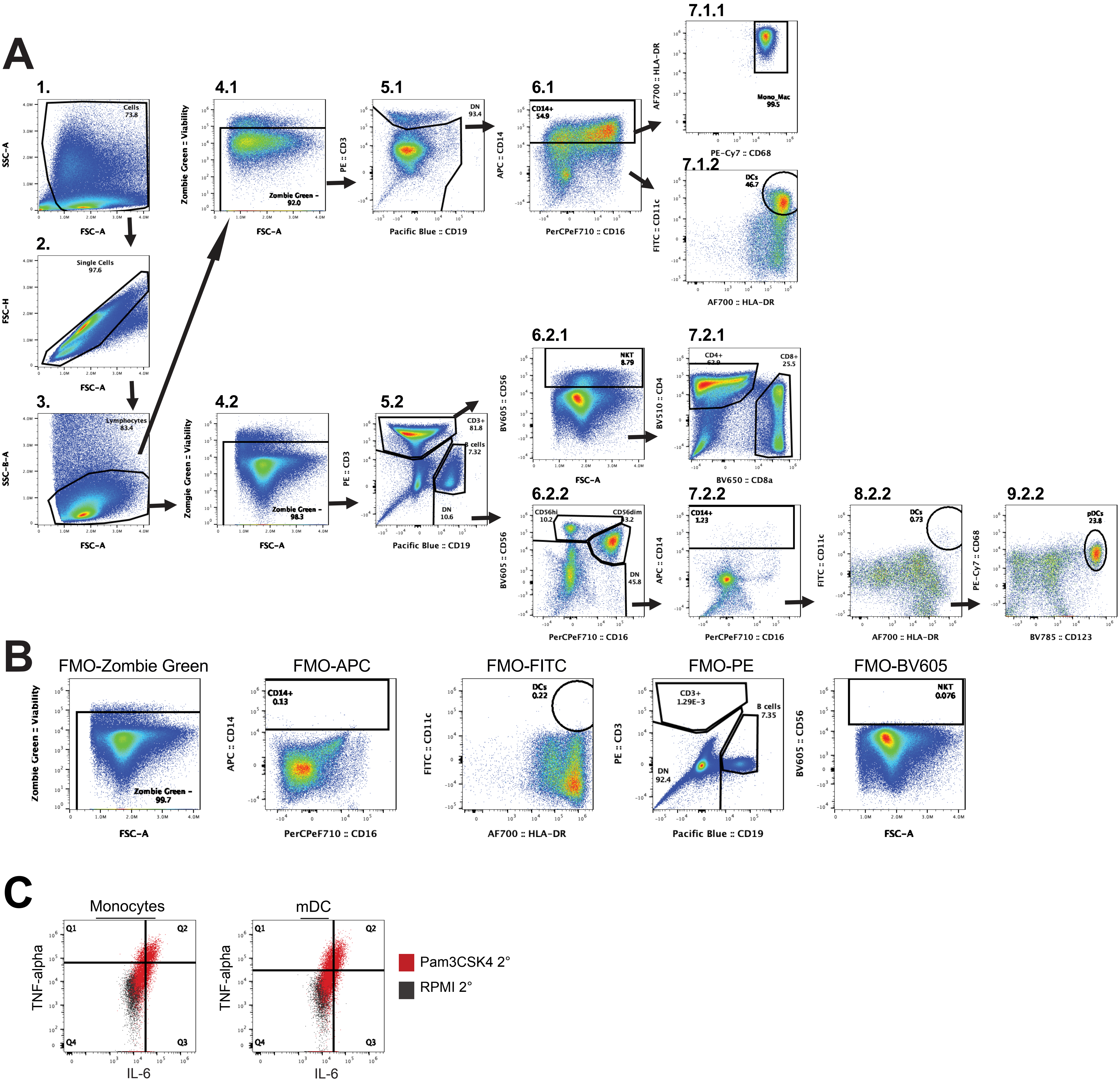
A
1.
Cells 73.8
SSC-A
FSC-A
2.
Single Cells 97.6
FSC-H
FSC-A
3.
Lymphocytes 83.4
SSC-B-A
FSC-A
4.1
Zombie Green :: Viability
Zombie Green – 92.0
FSC-A
4.2
Zomgie Green :: Viability
Zombie Green – 98.3
FSC-A
5.1
DN 93.4
PE :: CD3
Pacific Blue :: CD19
5.2
CD3+ 81.8
B cells 7.32
DN 10.6
PE :: CD3
Pacific Blue :: CD19
6.1
CD14+ 54.9
APC :: CD14
PerCPeF710 :: CD16
7.1.1
Mono_Mac 99.5
AF700 :: HLA-DR
PE-Cy7 :: CD68
7.1.2
DCs 46.7
FITC :: CD11c
AF700 :: HLA-DR
6.2.1
NKT 8.79
BV605 :: CD56
FSC-A
7.2.1
CD4+ 62.9
CD8+ 25.5
BV510 :: CD4
BV650 :: CD8a
6.2.2
CD56hi 10.2
CD56dim 43.2
DN 45.8
BV605 :: CD56
PerCPeF710 :: CD16
7.2.2
CD14+ 1.23
APC :: CD14
PerCPeF710 :: CD16
8.2.2
DCs 0.73
FITC :: CD11c
AF700 :: HLA-DR
9.2.2
pDCs 23.8
PE-Cy7 :: CD68
BV785 :: CD123
B
FMO-Zombie Green
Zombie Green :: Viability
Zombie Green – 99.7
FSC-A
FMO-APC
CD14+ 0.13
APC :: CD14
PerCPeF710 :: CD16
FMO-FITC
DCs 0.22
FITC :: CD11c
AF700 :: HLA-DR
FMO-PE
CD3+ 1.29E-3
B cells 7.35
DN 92.4
PE :: CD3
Pacific Blue :: CD19
FMO-BV605
NKT 0.076
BV605 :: CD56
FSC-A
C
Monocytes
mDC
Q1
Q2
Q3
Q4
TNF-alpha
IL-6
Pam3CSK4 2°
RPMI 2°

Supplementary Figure 2: Cytek Aurora Cell Phenotyping Panel

(A) Gating strategy to determine nine distinct cell types in trained PBMC. (B) Fluorescence-Minus-One (FMO) controls used to determine fluorescent staining thresholds. C) Overlays of indicated cell populations from iRBC-trained PBMC with RPMI as a secondary stimulus (Black) or with Pam3CSK4 as a secondary stimulus (Red).

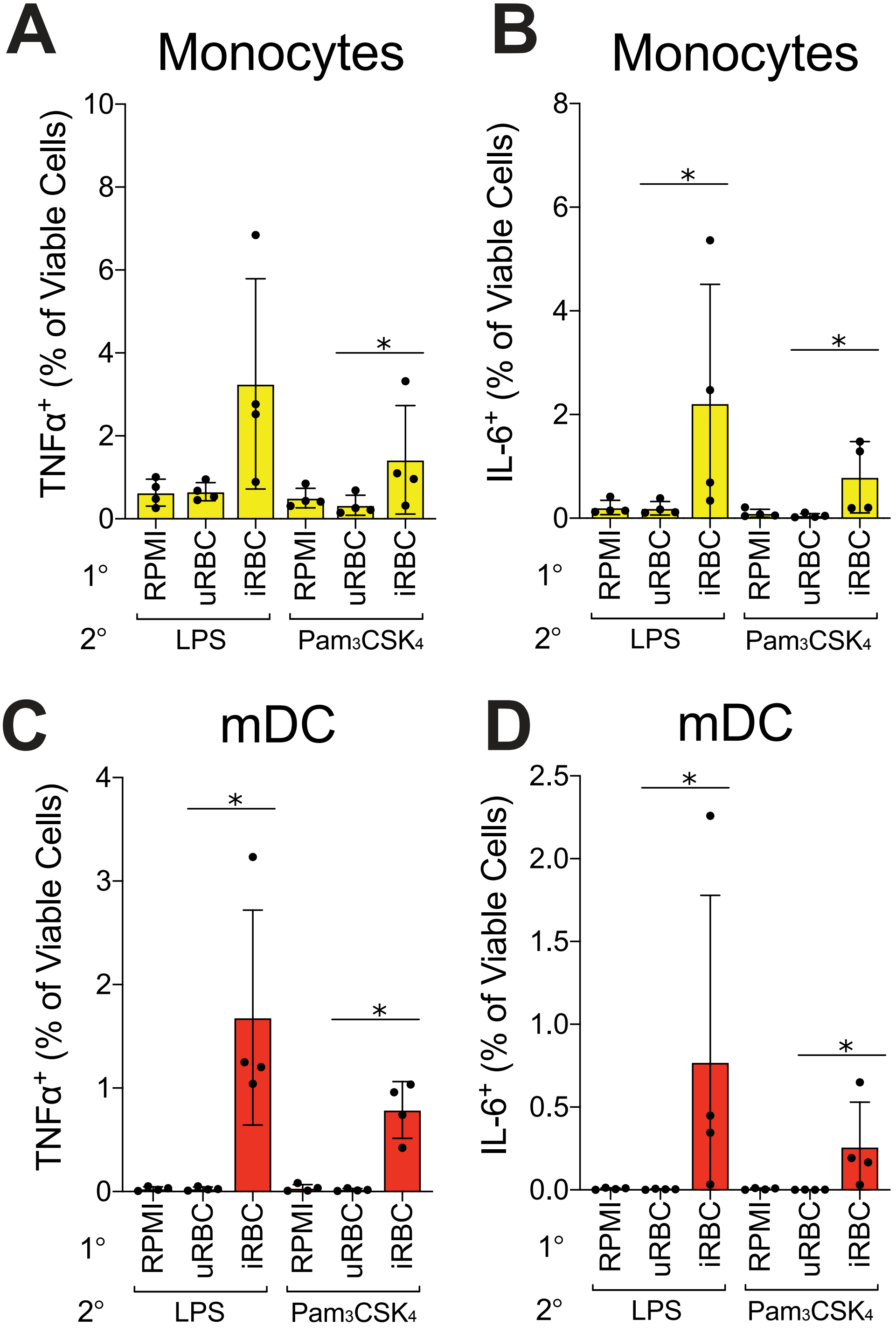

A
Monocytes
TNFα+ (% of Viable Cells)
10
8
6
4
2
0
*
1°
RPMI
uRBC
iRBC
RPMI
uRBC
iRBC
2°
LPS
Pam3CSK4
B
Monocytes
IL-6+ (% of Viable Cells)
8
6
4
2
0
*
*
1°
RPMI
uRBC
iRBC
RPMI
uRBC
iRBC
2°
LPS
Pam3CSK4
C
mDC
TNFα+ (% of Viable Cells)
4
3
2
1
0
*
*
1°
RPMI
uRBC
iRBC
RPMI
uRBC
iRBC
2°
LPS
Pam3CSK4
D
mDC
IL-6+ (% of Viable Cells)
2.5
2.0
1.5
1.0
0.5
0.0
*
*
1°
RPMI
uRBC
iRBC
RPMI
uRBC
iRBC
2°
LPS
Pam3CSK4

Supplementary Figure 3: Monocyte and mDC cytokine frequency

Monocytes and mDCs from trained PBMC restimulated with LPS or Pam_3CSK_4 for 5 hours. Frequency of total viable cells for monocytes producing TNFα (A) or IL-6 (B). Frequency of total viable cells for mDC producing TNFα (C) or IL-6 (D). N=4; error bars represent mean $\pm$ S.D. $*p \leq 0.05$.

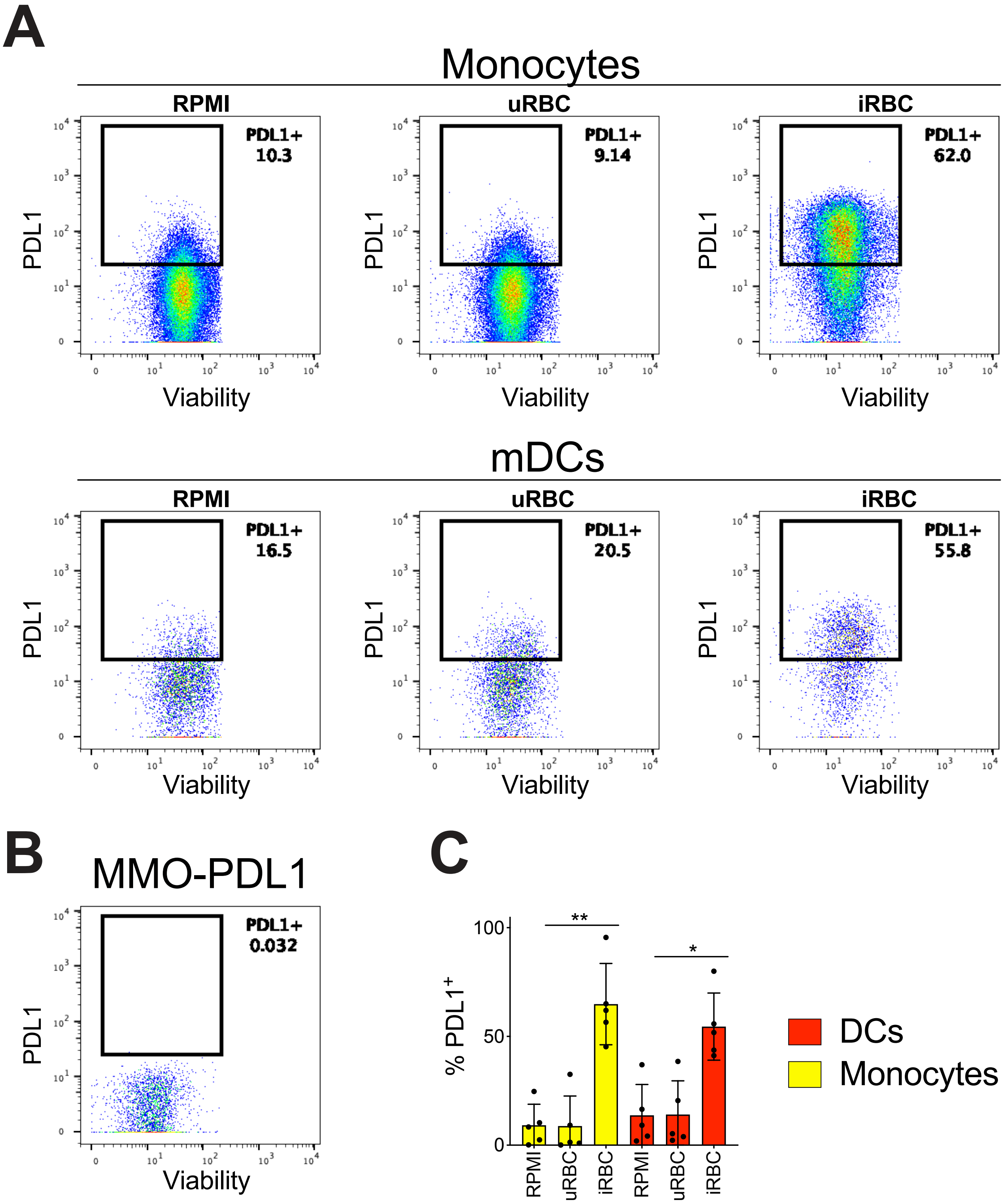

A
Monocytes
RPMI
uRBC
iRBC
PDL1+ 10.3
PDL1+ 9.14
PDL1+ 62.0
PDL1
Viability
mDCs
RPMI
uRBC
iRBC
PDL1+ 16.5
PDL1+ 20.5
PDL1+ 55.8
PDL1
Viability
B
MMO-PDL1
PDL1+ 0.032
PDL1
Viability
C
% PDL1+
100
50
0
**
*
RPMI
uRBC
iRBC
RPMI
uRBC
iRBC
DCs
Monocytes

Supplementary Figure 4: PDL1 is upregulated on Monocytes and mDCs in response to iRBC stimulation

CyTOF of PBMC stimulated with RPMI, uRBC or iRBC for 12 hours. (A) PDL1 surface expression on monocytes and mDCs from a representative donor. (B) Metal-Minus-One (MMO) of PDL1. (C) Frequency of PDL1 surface expression for monocytes and mDC. N=5; error bars represent mean $\pm$ S.D. * $p \leq 0.05$, ** $p \leq 0.01$.

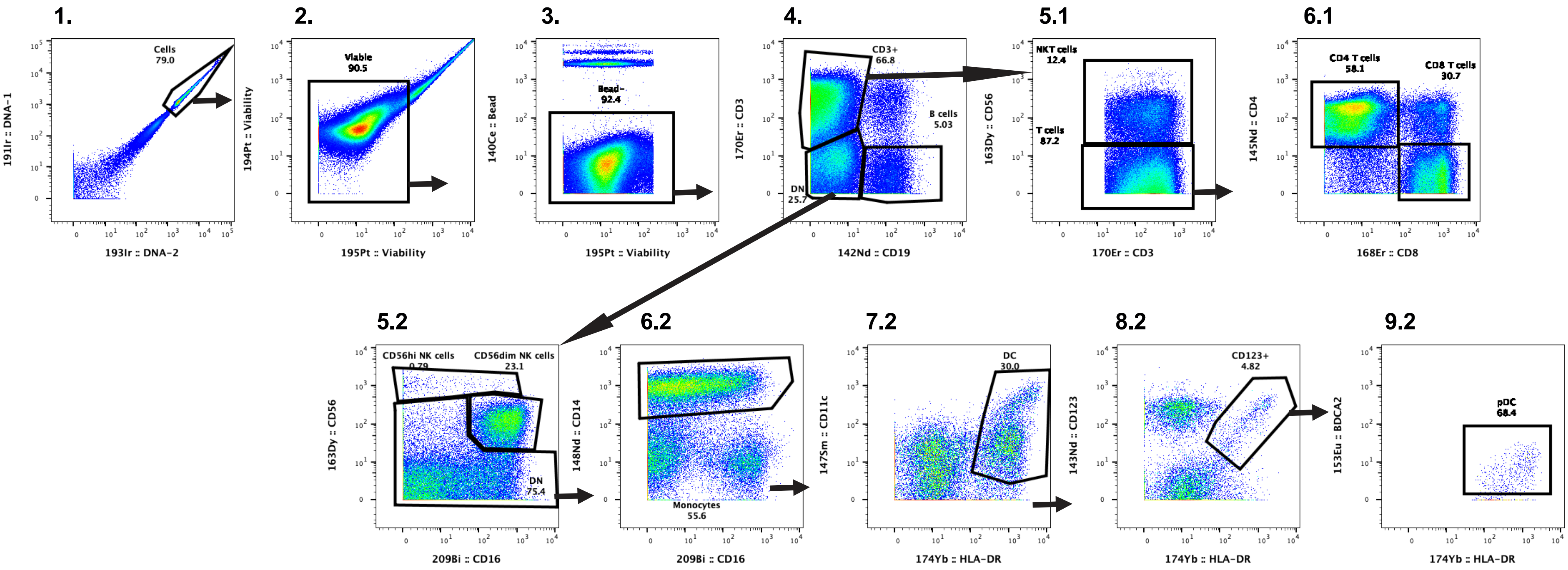
1.
Cells
79.0
191Ir :: DNA-1
193Ir :: DNA-2
2.
Viable
90.5
194Pt :: Viability
195Pt :: Viability
3.
Bead-
92.4
140Ce :: Bead
195Pt :: Viability
4.
CD3+
66.8
B cells
5.03
DN
25.7
170Er :: CD3
142Nd :: CD19
5.1
NKT cells
12.4
T cells
87.2
163Dy :: CD56
170Er :: CD3
6.1
CD4 T cells
58.1
CD8 T cells
30.7
145Nd :: CD4
168Er :: CD8
5.2
CD56hi NK cells
0.79
CD56dim NK cells
23.1
DN
75.4
163Dy :: CD56
209Bi :: CD16
6.2
Monocytes
55.6
148Nd :: CD14
209Bi :: CD16
7.2
DC
30.0
147Sm :: CD11c
174Yb :: HLA-DR
8.2
CD123+
4.82
143Nd :: CD123
174Yb :: HLA-DR
9.2
pDC
68.4
153Eu :: BDCA2
174Yb :: HLA-DR

Supplementary Figure 5: CyTOF cell phenotyping panel

Gating strategy to determine nine distinct cell types in PBMC stimulated for 12 hours with RPMI, uRBC or iRBC, and measure IFNγ production. Arrows indicate populations that are subgated on.

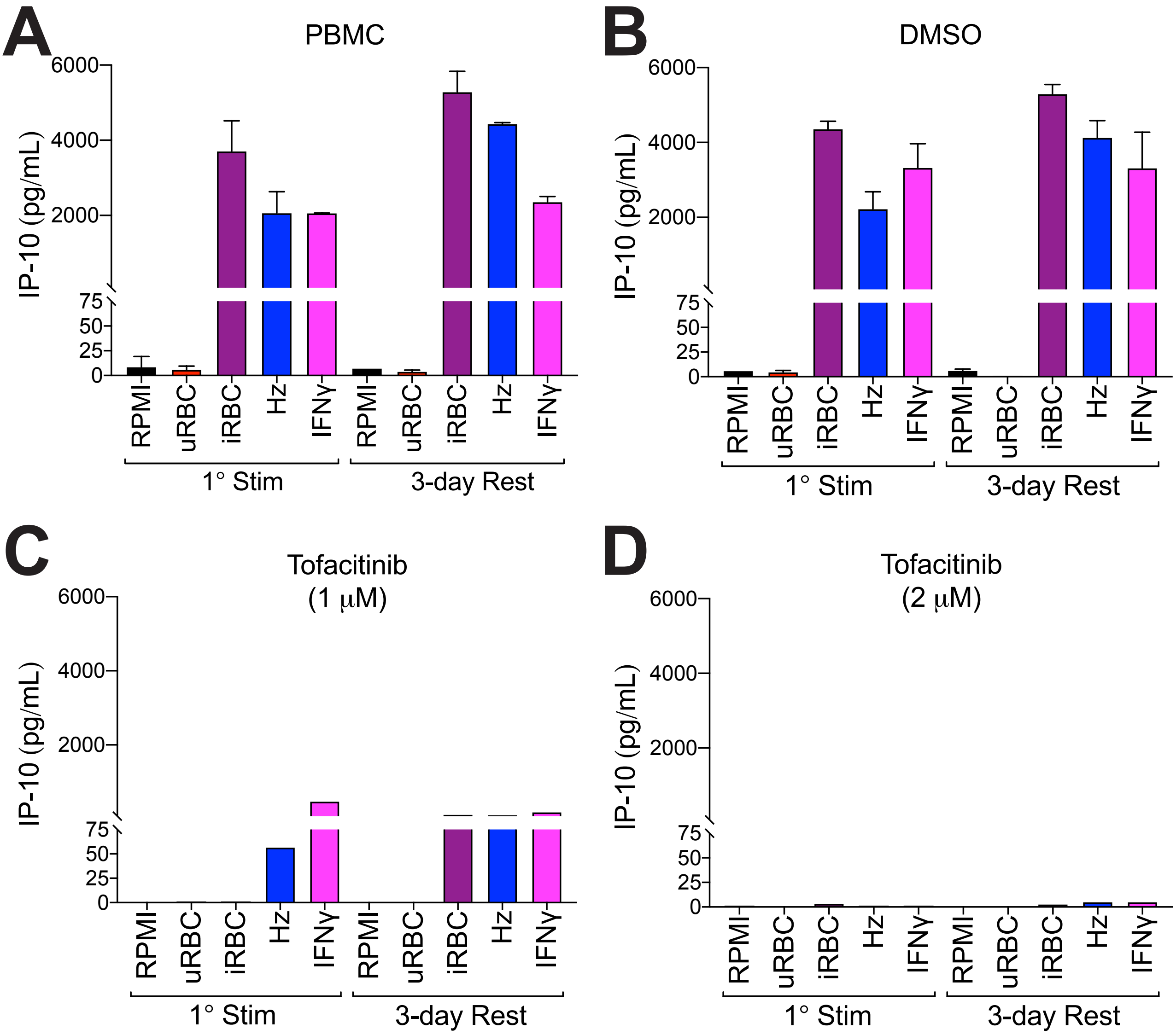
A
PBMC
B
DMSO
C
Tofacitinib
(1 μM)
D
Tofacitinib
(2 μM)
IP-10 (pg/mL)
6000
4000
2000
75
50
25
0
RPMI
uRBC
iRBC
Hz
IFNγ
1° Stim
3-day Rest

Supplementary Figure 6: Tofacitinib blocks IFNγR-mediated induction of IP-10/CXCL10

(A-D) IP-10 ELISA of supernatants from PBMC during primary training stimulus, and 3-day rest period. (A) PBMC. (B) PBMC treated with DMSO solvent during primary stimulus and 3-day rest. (C) PBMC treated with 1μM Tofacitinib during primary stimulus and 3-day rest. (D) PBMC treated with 2μM Tofacitinib during primary stimulus and 3-day rest.

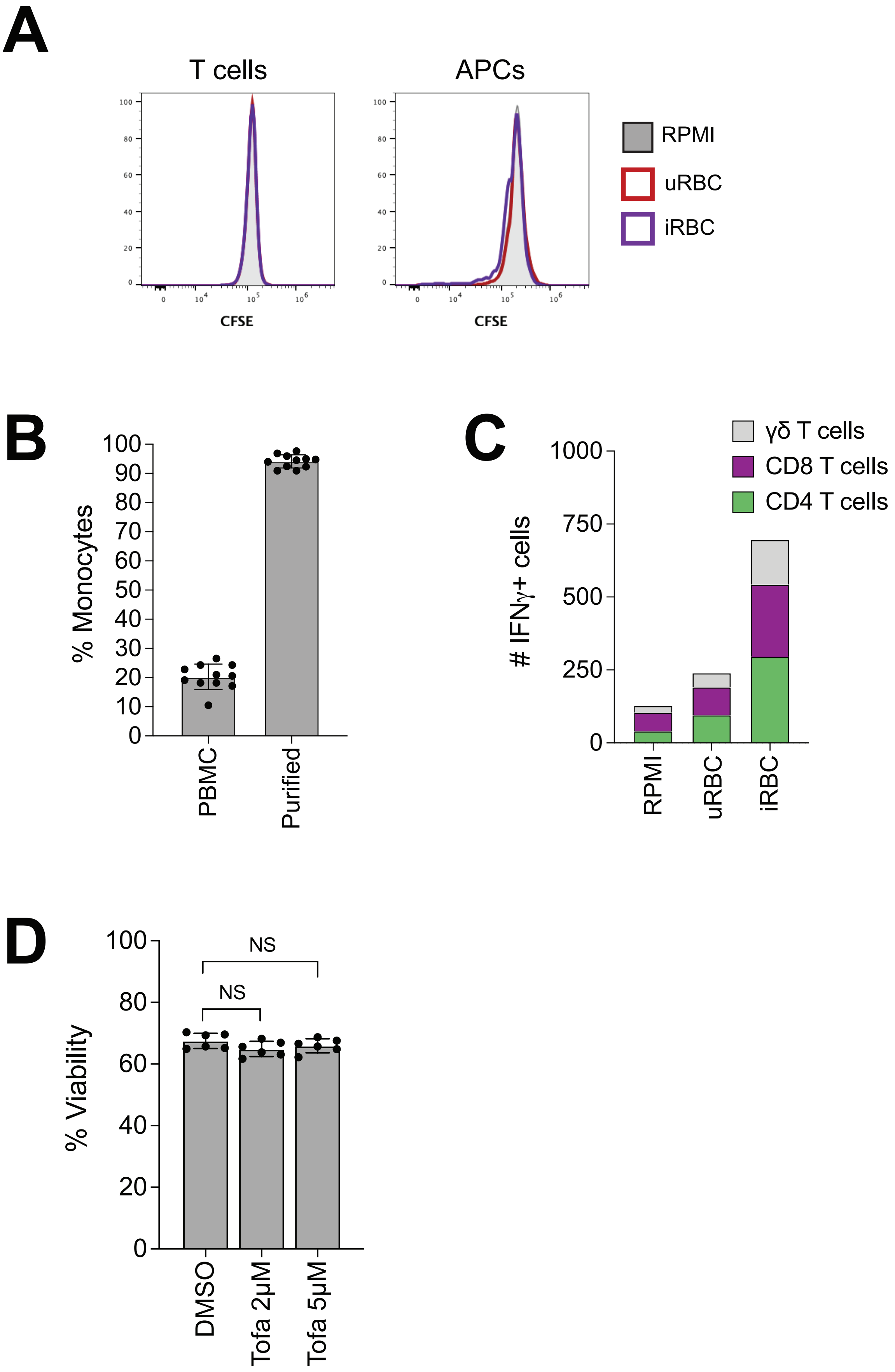
A
T cells
APCs
100
80
60
40
20
0
CFSE
RPMI
uRBC
iRBC
B
% Monocytes
100
90
80
70
60
50
40
30
20
10
0
PBMC
Purified
C
IFNγ+ cells
1000
750
500
250
0
γδ T cells
CD8 T cells
CD4 T cells
RPMI
uRBC
iRBC
D
% Viability
100
80
60
40
20
0
NS
NS
DMSO
Tofa 2μM
Tofa 5μM

Supplementary Figure 7:

(A) PBMC were labeled with CFSE and then trained with RPMI, uRBC or iRBC for 24 hours, and then rested 3 days. Cells were analyzed for proliferation by CFSE in T cells (CD3+) and in antigen-presenting cells (APCs) (HLA-DR+). Experiment is representative of 3 replicates (N=3 donors). (B) PBMC were enriched for monocytes using magnetic assisted cell sorting. The frequency of Monocytes was assessed by flow cytometry in the PBMC and the purified monocytes. N=11 Error bars represent Mean $\pm$ S.D. (C) PBMC were stimulated for 12 hours and assessed for IFNγ production by intracellular cytokine staining in gamma/delta T cells (CD3+gdTCR+), CD8 T cells (CD3+CD8+) and CD4 T cells (CD3+CD4+). N=4. (D) PBMC were treated with DMSO or Tofacitinib at indicated concentrations for 24 hours, and then viability was assessed using a fixable viability dye. N=6 Error bars represent Mean $\pm$ S.D.
